# Identification of transcription factor binding sites using ATAC-seq

**DOI:** 10.1186/s13059-019-1642-2

**Published:** 2019-02-26

**Authors:** Zhijian Li, Marcel H. Schulz, Thomas Look, Matthias Begemann, Martin Zenke, Ivan G. Costa

**Affiliations:** 1 Institute for Computational Genomics, Joint Research Center for Computational Biomedicine, RWTH Aachen University Medical School, Aachen, 52074 Germany; 2 Department of Cell Biology, Institute of Biomedical Engineering, RWTH Aachen University Medical School, Aachen, 52074 Germany; 3 Cluster of Excellence for Multimodal Computing and Interaction, Saarland Informatics Campus, Saarland University, Saarbrücken, Germany; 4 Computational Biology & Applied Algorithmics, Max Planck Institute for Informatics, Saarbrücken, Germany; 5 Institute for Cardiovascular Regeneration, Goethe University, Frankfurt am Main, Germany; 6 German Centre for Cardiovascular Research (DZHK), Partner site RheinMain, Frankfurt am Main, Germany; 7 Helmholtz Institute for Biomedical Engineering, RWTH Aachen University, Aachen, Germany; 8 Institute of Human Genetics, RWTH Aachen University Medical School, Aachen, Germany

**Keywords:** Computational footprinting, Open chromatin, ATAC-seq, Cleavage bias

## Abstract

Transposase-Accessible Chromatin followed by sequencing (ATAC-seq) is a simple protocol for detection of open chromatin. Computational footprinting, the search for regions with depletion of cleavage events due to transcription factor binding, is poorly understood for ATAC-seq. We propose the first footprinting method considering ATAC-seq protocol artifacts. HINT-ATAC uses a position dependency model to learn the cleavage preferences of the transposase. We observe strand-specific cleavage patterns around transcription factor binding sites, which are determined by local nucleosome architecture. By incorporating all these biases, HINT-ATAC is able to significantly outperform competing methods in the prediction of transcription factor binding sites with footprints.

**Electronic supplementary material:**

The online version of this article (10.1186/s13059-019-1642-2) contains supplementary material, which is available to authorized users.

## Background

DNase-I hypersensitive sites sequencing (DNase-seq; [1–4]) and Assays for Transposase-Accessible Chromatin sequencing (ATAC-seq; [5, 6]) are two widely used protocols for genome-wide identification of open chromatin. DNase-seq and ATAC-seq are based on the use of cleavage enzymes (DNase-I and Tn5, respectively), which recognize and cleave DNA in open chromatin regions. Sequencing and the alignment of reads from these fragments allows the detection of open chromatin by identifying genomic intervals with many reads [1, 2]. However, the presence of transcription factors (TFs) bound to the DNA prevents the enzyme from cleavage in an otherwise nucleosome-free region. This leaves small regions, referred to as footprints, where read coverage suddenly drops within peak regions of high coverage.

Computational methods scanning open chromatin profiles to find footprints have been shown to predict transcription factor binding sites (TFBS) with high accuracy in DNase-seq data [7, 8]. Among others, computational footprinting has been used to detect the regulatory lexicon of several cell types [9, 10], to measure the effects of genetic variants in TF binding [11] and to assess changes in the activity of TFs, e.g., during inflammatory responses [12] or fasting conditions [13]. Computational footprinting, which only requires a single open chromatin experiment per cell of interest, is a powerful tool to study regulatory processes.

ATAC-seq has several experimental advantages over DNase-seq: it requires fewer cells (50.000 to single cells) and is less laborious [5, 6]. Not surprisingly, the number of ATAC-seq-based studies deposited in Gene Expression Omnibus is twelve times higher than the number of DNase-seq-based studies in the last year (366 ATAC-seq vs. 29 DNase-seq)^1^. There is also two times more ATAC-seq samples than DNase-seq samples per study, confirming that its experimental simplicity makes it a good choice for studies with large sample size, for example in clinical settings [14]. However, computational footprinting is still poorly explored in ATAC-seq data. The single study contrasting ATAC-seq and DNase-seq shows that ATAC-seq footprints have inferior accuracy than DNase-seq footprints [15]. It was also reported that ATAC-seq average footprint profiles are not so well defined as average footprint profiles from DNase-seq [11]. However, all the work with footprinting in ATAC-seq so far [5, 15, 16] used computational methods tailored to DNase-seq data and ignored characteristics intrinsic to the ATAC-seq protocol.

A possible reason for the lower performance of ATAC-seq footprinting might be the cleavage enzyme Tn5 itself, which has a large (17bp) “Tn5 motif” [5, 17] and a complex cleavage mechanism requiring a Tn5 dimer for action. The large size of the Tn5 dimer makes cleavage events dependent on structural features of the neighboring proteins (TFs or histones) and on the size of accessible DNA [18]. Cleavage events in small linker DNA between nucleosomes are possible, but less likely than cleavage of fragments from active regulatory regions [5]. Importantly, the DNA binding preferences of enzymes cause sequence-specific cleavage bias. Thus, computational bias correction is an important aspect of the analysis of DNase-seq [19, 20] and ATAC-seq data [21]. Some work uses position weight matrices (PWMs), which assume independence between positions, to model DNase-seq bias [22]. However, most bias correction methods infer bias estimates using *k*-mer sequences around the start of aligned reads, by estimating the probability of finding a *k*-mer at read start sites against occurrences in the genome [19]. For DNase-seq, a *k* equal to 6 was frequently used [8, 11, 19, 20, 23]. This method requires the estimation of a multinomial distribution and is likely to suffer from overfitting for large *k*-mers [24]. Alternatively, position dependency models (PDMs) allow flexibility in the type of dependencies being modeled [25, 26]. They have been shown to overcome the problem of overfitting in modeling protein-DNA binding preferences. We are unaware of methods exploring effects of the local chromatin structure in ATAC-seq or the use of PDMs for modeling the bias of cleavage enzymes.

Here, we propose HINT-ATAC, which is the first footprinting method dealing with the characteristics of the ATAC-seq protocol. First, we propose the use of a probabilistic PDM based on sparse local inhomogeneous mixtures (SLIM) models for the correction of cleavage bias [26] and evaluate it for both ATAC-seq and DNase-seq protocols. Second, we model a novel observation that ATAC-seq cleavage events show a strand bias, which is associated to the number of nucleosomes in ATAC-seq fragments. HINT-ATAC, which is based on hidden Markov models, uses strand-specific, nucleosome-size decomposed, and bias-corrected signals to identify footprints. We show that HINT-ATAC significantly improves the recovery of footprints supported by TF ChIP-seq data [8, 27] from ENCODE cell lines [9]. Moreover, HINT-ATAC footprints have similar predictive accuracy using either ATAC-seq or DNase-seq protocols. Finally, as an example of practical application of footprint analysis, we use HINT-ATAC to detect TFs associated with immune dendritic cell (DC) specification.

## Results

### The transposase Tn5 has a complex cleavage bias

**Fig. 1:**
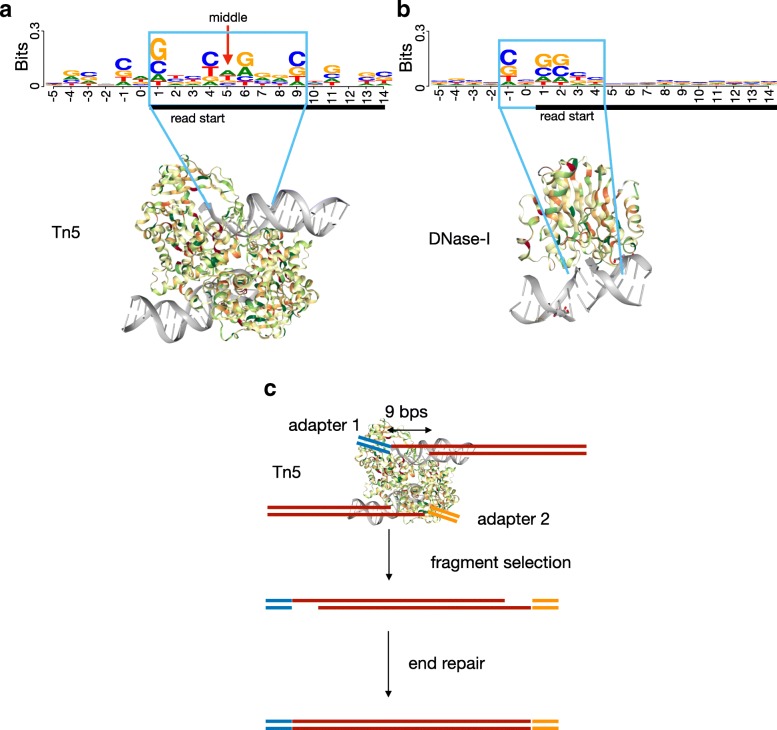
Cleavage enzymes of ATAC-seq and DNase-seq. Sequence motif relative to aligned read starts after cleavage with Tn5 and DNase I enzymes on naked DNA ATAC-seq (**a**) and DNase-seq (**b**) experiments. Position 1 corresponds to the start position of the ATAC/DNase-seq read. The size of the motifs is reflected by the structural protein contacts of Tn5 and DNase-I (Protein Data Bank entries 1MM8 and 2DNJ). **c** Tn5 inserts adapters in both DNA ends. Moreover, DNA is cleaved into two 9 bps single ends, which are later repaired in the ATAC-seq protocol

Cleavage bias is caused by the preference of enzymes to cleave particular DNA sequences [19] as indicated by the motifs around the start sites of DNase-seq and ATAC-seq reads (Fig. 1a, b). Motifs are similar for distinct ATAC-seq libraries and protocol variations such as standard [5], Omni-[6], and Fast-ATAC [28] (Additional file 1: Figure S1). The Tn5 dimer cleaves the DNA by inserting two distinct adapters in the DNA fragment ends. Cleavage leaves two 9 bps single-strand DNA ends that are later extended in the ATAC-seq protocol (Fig. 1c). The fact that Tn5 works as a dimer, where two Tn5 proteins bind to the DNA in reverted orientations, causes the large (9–13 bps) palindromic Tn5 motif (Fig. 1a). Moreover, the motif is centered around position + 5 relative to the read start, which represents the middle position of the Tn5 cleavage event. In contrast, DNase-I leaves a short motif close to the start of reads in DNase-seq experiments (Fig. 1b).

### Position dependency models improve cleavage bias correction

We evaluate here the use of position dependency models (PDM) to estimate the bias of Tn5 cleavage events. HINT-ATAC considers the fifth base of ATAC-seq reads as the cleavage event as in [5]. A PDM learns relevant dependencies from the data and is less likely to overfit than *k*-mer-based approaches, when large sequences need to be considered. We compare the performance of PDMs to *k*-mer or PWM-based bias correction, the two methods previously used in the literature (see the “Method” section). This includes an analysis of the optimal word size *k* necessary to capture cleavage bias for both Tn5 and DNase-I. Cleavage signals obtained by distinct correction methods (and uncorrected signals) are given as input for the footprinting method HINT [7] (Additional file 1: Figure S2). We then evaluate the recovery of footprints with binding sites supported by ChIP-seq peaks on 32 TFs from the GM12878 cell (training dataset). For this, we calculate the area under precision recall curve (AUPR) and the area under receiver operating characteristics curve (AUC) for distinct false positive rates (1%, 10 *%*, and 100%) for each TF as in [8]. A final ranking score is obtained by combining the ranking of a method for each of the six statistics. A higher ranking score indicates higher recovery of ChIP-seq supported footprints.

**Fig. 2:**
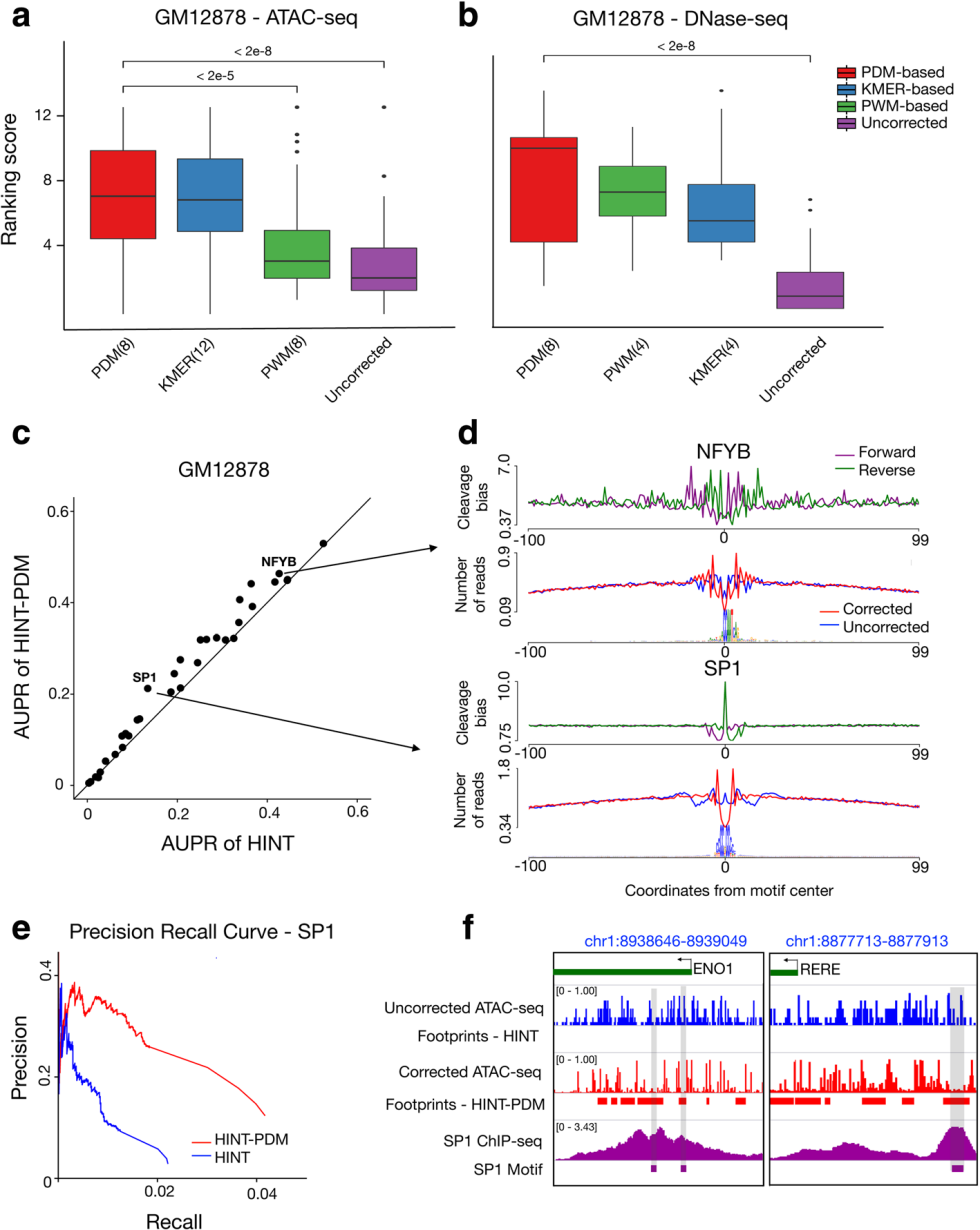
Strategies for cleavage bias correction. Comparison of bias estimation methods in standard ATAC-seq (**a**) and DNase-seq (**b**) on 32 TF ChIP-seq data sets from GM12878 cells. The *y*-axis denotes the ranking score, where higher values indicate higher recovery of footprints supported by TF ChIP-seq peaks. Numbers after methods names (*x*-axis) indicate optimal word size (*k*). *p* values are based on the Friedman-Nemenyi test (see Additional file 1: Table S1–S12 for complete results). **c** The scatter plot contrasting AUPR of HINT with PDM-based estimation with 8-mers (*y*-axis) and HINT without bias correction (*x*-axis) in GM12878 cells. **d** Bias estimates and average ATAC-seq signals centered around NFYB and SP1 motifs supported by a ChIP-seq peaks in GM12878 cells. **e** Precision-recall curve also supports the improvement in prediction of SP1 ChIP-Seq supported binding sites with cleavage bias correction. **f** ATAC-seq cleavage signals and footprint predictions with (HINT-PDM) and without (HINT) bias correction in two selected genomic regions. Footprint predictions on bias-corrected signals match SP1 motifs supported by ChIP-seq peaks, while no footprints are predicted in uncorrected ATAC-seq due to the presence of cleavage sites within the SP1 motif

The comparative analysis indicates that PDMs are best for footprint detection in all evaluated libraries with the exception of Omni ATAC-seq, where *k*-mer and PDM tied first (Fig. 2a, b, Additional file 1: Figure S4). One important question is the robustness of methods when estimated on libraries with different sequencing depths. Therefore, we perform random under-sampling of an ATAC-seq library by decreasing its size from 70 to 35 million reads. We observe that the PDM is ranked first when considering only 75% or 50% of Omni-ATAC-seq reads (Additional file 1: Figure S4). Moreover, bias estimates from PDMs remain highly similar after under-sampling, while *k*-mer estimates show increasing variance with less reads (Additional file 1: Figure S5). Another relevant question is the size of the sequence (*k*), which needs to be considered for capturing cleavage bias. Interestingly, smaller sequences (4–8) are selected for DNase-seq data, while larger sequences (8–12) are best for ATAC-seq protocols (Fig. 2a, b).

These results fit with the observation that the most conserved positions in the Tn5 motif, i.e., positions 1 and 9 in Fig. 1a, are farther away than in the DNase-I motif, i.e., positions −1 and 2. As a consequence, a larger sequence size is necessary to correct the Tn5 bias. We also observe that the distribution of cleavage bias estimates is more dispersed in ATAC-seq than in DNase-seq (Additional file 1: Figure S6), which indicates more extreme bias for particular sequences for ATAC-seq libraries. Another important question is the dependencies between sequence positions, which are observed on parameters learned by PDMs or by a statistical test proposed in [29]. While neighboring (first order) dependencies are most relevant for both Tn5 and DNase-I enzymes, a few higher order dependencies are relevant for ATAC-seq (Additional file 1: Figure S7). These results support the fact that PDMs, which learn relevant dependencies from the data, are more suitable for modeling the bias of Tn5. We adopt the use of PDM with *k*=8 as standard in HINT-ATAC in all experiments below.

Another relevant question is the use of ATAC-seq and DNase-seq libraries performed on naked DNA, which were previously proposed as control libraries for measurement of cleavage bias [30]. However, results indicate that bias estimates based on GM12878 cells have higher ranks than the use of naked DNA (Additional file 1: Figure S8). This is also supported by the similarity of bias estimates on several ATAC-seq libraries, where bias estimates based on the naked DNA group apart from bias obtained in the libraries themselves (Additional file 1: Figure S8). Given that cleavage bias varies in distinct degrees for each library, these results support the use of bias estimates based on reads from the ATAC-seq library at hand.

### Cleavage bias estimates and TF-specific impact

It was previously shown that the impact of cleavage bias correction is TF-specific [8, 19, 30], i.e., TFs with motifs similar to the enzyme motif will not leave clear footprints. Therefore, we compare the AUPR of 32 individual TFs predicted with bias-corrected and uncorrected signals (Fig. 2c). Most of the TFs (29 out of 32) have an increase in AUPR, while the AUPR decrease for 3 TFs is marginal (average of 0.003; Additional file 1: Figure S9). As expected, TFs with the highest increase in AUPR (NFYB and Sp1 Fig. 2d, e) have depletion of ATAC-seq cleavage sites around their binding sites after bias correction. Moreover, ATAC-seq profiles in individual genomic locations also support the advantage of cleavage bias correction in the detection of footprints (Fig. 2f).

### Incorporation of nucleosome density and strand information improves footprinting

**Fig. 3:**
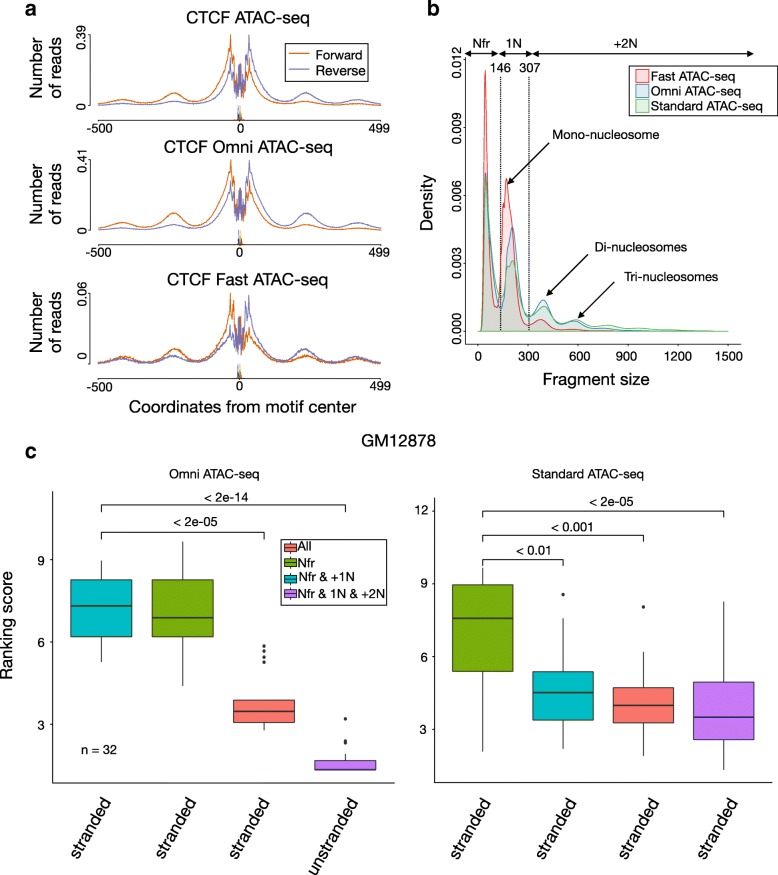
Local nucleosome architecture and footprints. **a** Cleavage profiles around CTCF ChIP-seq peaks indicate strand-specific cleavage preference left/right of the TF binding site for distinct ATAC-seq protocols in GM12878 cells. Smaller peaks away from the center represent linker regions between histones. **b** Fragment size distribution for ATAC-seq protocols on GM12878 cells indicates clear peaks representing fragments with particular numbers of nucleosomes. Local minimum values were used to define nucleosome-free fragments Nfr, fragments with one nucleosome 1N and fragments with one or more +2N nucleosomes. **c** Comparison of HINT-ATAC models with distinct nucleosome decomposition strategies of Omni ATAC-seq (left) and standard ATAC-seq (right) on GM12787 cells. A higher ranking score (*y*-axis) indicates highest recovery of ChIP-seq supported binding sites. Labels in the *x*-axis indicate if strand information is used by the model. *p* values are based on the Friedman-Nemenyi test

We observe strong strand-specific patterns on average ATAC-seq profiles around CTCF ChIP-seq peaks (Fig. 3a), which was not reported before for ATAC-seq data. This is also observed in individual genomic loci with CTCF-binding sites (Additional file 1: Figure S10). As strand specificity is particularly high in linker regions, we reason that it could be associated with the number of nucleosomes included in the ATAC-seq fragment. Sizes of ATAC-seq fragments, which can be estimated from paired-end sequencing libraries, have typical modal distributions associated to fragments with zero, one, two, or more nucleosomes [5]. We observe that distinct ATAC-seq protocols have slightly distinct fragment size distributions, which reflect their bias towards more (or less) nucleosome-containing fragments (Fig. 3b).

Therefore, we decompose ATAC-seq cleavage signals by only considering reads from nucleosome-free fragments or reads from fragments with a particular number of nucleosomes (Additional file 1: Figure S11). We evaluate the performance of HINT-ATAC by providing ATAC-seq signals with reads from distinct decomposition strategies (fragment sizes) as input and by varying the number of HMM states (see the “Method” section and Additional file 1: Figure S12). This includes considering all reads (all), nucleosome-free fragments (Nfr), nucleosome-free and nucleosome-containing fragments, (Nfr & +1N) and nucleosome-free, one nucleosome, and two or more nucleosomes fragments (Nfr & 1N & +2N). We further evaluate the use of strand-specific and non-strand specific signals, where the dimensions of input signals vary from 2 to 12^2^. HINT-ATAC models are evaluated on the prediction of 32 TFs in GM12878 cells (training dataset).

The comparative evaluation indicates that using Nfr is best for standard ATAC and Fast-ATAC protocols, while the combined use of nucleosome-free and nucleosome-containing signals (Nfr & +1N) is best for Omni-ATAC (Fig. 3c, Additional file 1: Figure S13). Moreover, optimal HMM models are always based on strand-specific signals. These results indicate the importance of considering fragment sizes and strand information for improving footprint detection in ATAC-seq data. From here on, we will use the optimal HMM configuration for each ATAC-seq protocol determined on the training dataset.

An inspection of the parameters of the Omni-ATAC HMM gives insights on how HINT-ATAC uses nucleosome decomposed cleavage signals to detect footprints (Additional file 1: Figure S14). This HMM includes states associated to the footprint, flanking regions left/right of the footprint and background regions. Interestingly, the left flanking state has high emission values for Nfr forward reads and +1N reverse reads, while the right flanking state has high emission values on Nfr reverse reads and +1N forward reads. This indicates the importance of strand-specific signals and the presence of reverted strand-specific cleavage patterns on reads from nucleosome-free and nucleosome-containing fragments around the footprint.

### Local nucleosome architecture and strand-specific ATAC-seq cleavage profiles

**Fig. 4:**
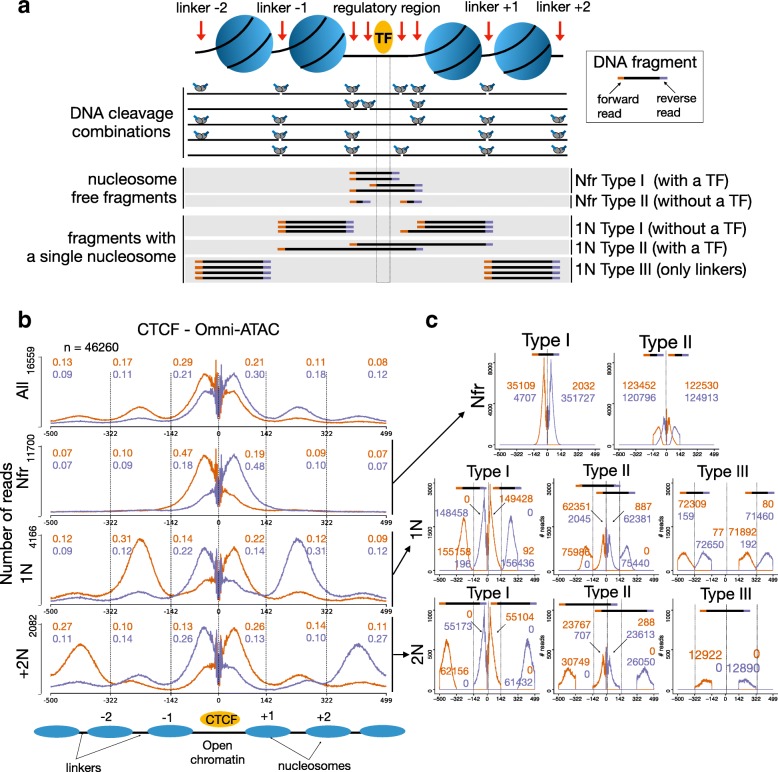
Nucleosome architecture and strand-specific cleavage profiles. **a** Tn5 digests open chromatin regions left/right of the TF binding (regulatory region) or in nucleosome linkers. Nucleosome-free fragments will generate reads with (Nfr type I) or without (Nrf type II) the TF bound to DNA. As sequencing is performed from the 5 ^′^ to 3 ^′^ ends, Nfr type I fragments will always generate forward reads on the left (orange) and reverse reads on the right (blue) relatively to the TF binding site. DNA fragments from 1N decomposition with a cleavage event in the regulatory region will either include (1N Type II) or not (1N Type I) a TF. 1N Type III are produced by cleavage events between two neighboring linkers. **b** Bias-corrected average cleavage profile around CTCF ChIP-seq peaks for Omni-ATAC in GM12878 cells for fragments with distinct number of nucleosomes. Strand bias can be estimated as the ratio of reads in forward (orange) and reverse (blue) around intervals between nucleosomes and CTCF. **c** Decomposition of Nfr, 1N and 2N fragments by types clarifies the origin of strand cleavage bias. Numbers in orange (blue) indicate amount of reads in the forward (reverse) strand at each interval

Previous results indicate that the combination of strand-specific signals and decomposition by nucleosome numbers significantly improves footprint prediction in all ATAC-seq protocols. To understand the mechanism behind strand bias, we define types of ATAC-seq cleavage events relative to the location of the TF binding site (Fig. 4a). Next, we measure the amount of the strand cleavage bias for distinct fragment sizes (All, Nfr, 1N and +2N) around distinct intervals near the TF binding site (Additional file 1: Figure S15). We observe in Fig. 4b that there are more forward reads left to CTCF binding in nucleosome-free fragments (forward/reverse ratio of 2.6), while there are more reverse reads left of CTCF for nucleosome-containing fragments (ratios of 0.63 for 1N and 0.5 for +2N). This bias is not so prominent when considering all reads together (ratio of 1.38 for All). We also observe high strand-specific bias in reads in linker regions, i.e., more forward reads in linkers −2 and −1.

To further understand these observations, we separate reads by the types as proposed in Fig. 4a and count read frequencies in distinct regions (Fig. 4c). Considering Nfr fragments, we observe that Nfr type I generates almost exclusively forward reads left of CTCF. As sequencing is performed in the 5’ to 3’ direction, Nfr type I reads will only give rise to forward reads left of CTCF, while type II reads will generate both forward and reverse reads left of CTCF. Moreover, ATAC-seq protocols disfavor very short fragments associated to Nfr type II (Fig. 3b). These two facts cause the presence of a large number of forward reads from Nfr fragments left of the TF binding site. Following a similar rationale, 1N type I fragments generate reverse strand reads left of CTCF, while 1N type II fragments generate forward reads left of CTCF. There is a higher number of 1N type I reads than 1N type II reads, as ATAC-seq protocols bias disfavours too long fragments (Fig 3b). 1N Type III reads are not relevant here, as their starting sites do not help the delineation of footprints. Equivalent patterns are also found in +2N reads. Similar strand bias for nucleosome decomposed signals are found in all ATAC-seq protocols and TFs (Additional file 1: Figure S17–S19), despite some variance in the distance between TF and linker regions [5].

### Comparative evaluation of footprinting methods in ATAC-seq data

Next, we evaluate the performance of HINT-ATAC and state-of-the-art footprinting methods using an independent dataset based on K562 and H1-ESC cells (in total 148 TFs). We use three footprinting methods (DNase2TF, PIQ, and Wellington), which performed best in a recent comparative study based on DNase-seq data [8], and DeFCoM, which was recently proposed for ATAC-seq data [15]. We have adapted Wellington and DeFCoM to evaluate them with PDM-based bias correction. As the baseline method, we include Tag Count (TC), which simply considers TFBSs inside peaks ranked by the number of reads after cleavage bias correction.

**Fig. 5:**
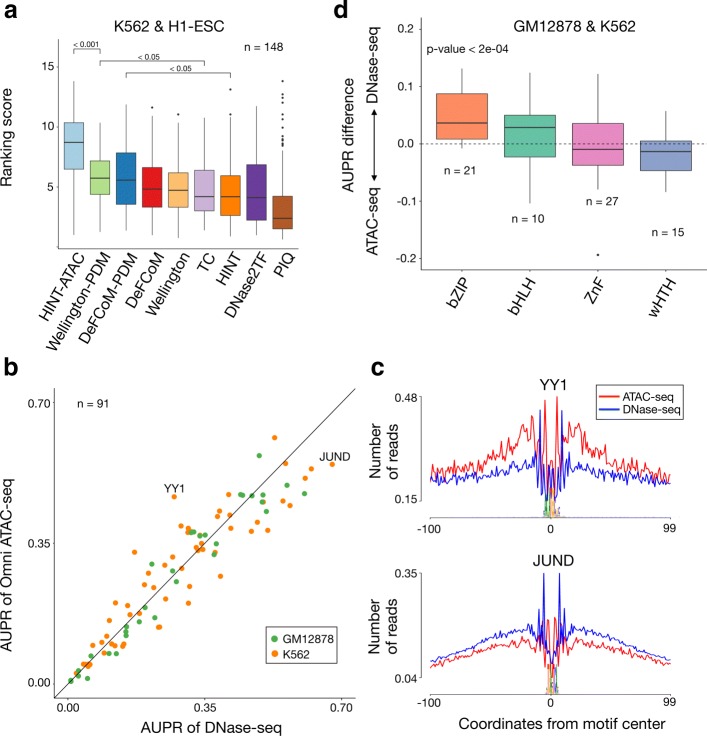
Competing methods and protocols comparison. **a** Comparative evaluation of HINT-ATAC, HINT, Wellington, DNase2TF, DeFCoM, and PIQ on the test dataset (H1-ESC and K562 cells). A higher ranking score indicates highest recovery of ChIP-seq supported binding sites. *p* values are based on the Friedman-Nemenyi test. We only show the significant *p* values of the top 3 methods (see Additional file 1: Table S23–S24 for complete results). **b** AUPR values of DNase-seq (DH) vs ATAC-seq (Omni) for 91 factors, of which 41 factors obtain higher AUPR using ATAC-seq. **c** The footprint profiles of two factors with the highest AUPR difference are shown. **d** Difference in AUPR of double-hit DNase-seq and Omni ATAC-seq by grouping TFs by transcription factor families as defined in JASPAR database. Only families with more than 10 TFs are shown, and *p* values are obtained with a *t* test (mean = 0)

As before, methods are evaluated with the ranking scores, which combine AUPR and AUC values for distinct false positive rates for each TF. A higher ranking score indicates higher recovery of ChIP-seq supported footprints. HINT-ATAC is the top ranked method followed by Wellington and DeFCoM using PDM bias correction. HINT-ATAC has statistically significant higher ranking than all evaluated methods, and Wellington-PDM has statistically significant higher ranking than TC (Fig. 5a). One interesting question is the independent importance of (1) PDM bias correction and (2) nucleosome decomposition in HINT-ATAC performance. We observe that the independent use of PDM bias correction or nucleosome decomposition improves the performance of HINT, while neither improvement is significantly better than the other (Additional file 1: Figure S20).

Concerning competing methods, ranking of Wellington and DeFCoM is improved with the use of PDM bias correction, but we observe no clear improvement when using Nfr reads (Additional file 1: Figure S20)^3^. The overall good performance of Wellington-PDM is likely due to its use of strand-specific signals. DeFCoM, which is based on a classifier, requires TF ChIP-seq data for training a model for each individual TF and is the only method requiring training on K562 and H1-ESC cells. Its performance is likely to decrease when training and predictions are performed across distinct cells. Taken together, these results confirm the fact that bias correction based on PDMs is crucial for prediction of ATAC-seq footprints and that HINT-ATAC has the best recovery of ChIP-seq supported footprints.

### Omni-ATAC-seq and DNase-seq are equivalent in the prediction of cell-specific transcription factor binding

There is a perception in the field that DNase-seq libraries are superior to ATAC-seq for computational footprinting [11, 15]. To address this, we compare the predictive performance of HINT-ATAC on distinct ATAC-seq protocols and DNase-seq of single-hit [31] and double-hit [32] protocols. We also analyze ATAC-seq experiments on distinct number of cells (bulk 50.000, 500, or single cells) [33]. To obtain a fair comparison, we first optimize HINT models for DNase-seq to consider strand-specific signals and the use of PDM-based correction (Additional file 1: Figure S13). Altogether, double-hit DNase-seq and Omni ATAC-seq are ranked best in all evaluated cells, while fast-ATAC-seq or ATAC-seq experiments based on 500 cells obtain the poorest results (Additional file 1: Figure S21). The lower performance of the former protocols is explained by their low quality indicators, i.e., fraction of reads insides peaks (FRIP) below 0.1 (Additional file 1: Figure S21).

Another limitation of ATAC-seq, which was previously discussed in the literature, is its lower coverage in enhancer regions [6, 34]. To inspect if this also impacts on the prediction of footprints in enhancers, we divide ChIP-seq peaks as being in a promoter and enhancer regions using chromHMM annotations [35] and evaluate AUPR on both subsets. Indeed, we observe that footprints based on standard ATAC-seq have lower performance in enhancer regions relative to footprints based on DNase-seq data. However, no such difference is observed using Omni-ATAC-seq (Additional file 1: Figure S22). This indicates that improvements in the Omni-ATAC-seq protocol have a positive impact on the prediction of TFBSs in enhancers.

Finally, we inspect if one of the protocols is able to predict more accurately the binding of particular TFs. We observe that TFs with higher AUPR on ATAC-seq have more ATAC-seq cleavage sites surrounding the binding site and vice-versa, as exemplified by YY1 and JunD (Fig. 5b, c and Additional file 1: Figure S23). Interestingly, grouping of TFs by family indicates that DNase-seq obtains higher AUPR for bZIP and helix-loop-helix families (Fig. 5d and Additional file 1: Figure S24). This suggests that structural features shared by TF families negatively affect Tn5 cleavage.

### HINT-ATAC finds relevant transcription factors for dendritic cell specification

To demonstrate the performance of HINT-ATAC, we use HINT-ATAC footprints for detecting TFs in dendritic cells (DC), a specialized immune cell type involved in immunity and tolerance induction [36]. In short, we use a two-step culture system [37, 38] to differentiate multipotent progenitors (MPP) from mouse bone marrow into common DC progenitors (CDP). CDP are then further differentiated into classical DC type1 and type2 (cDC1 and cDC2, respectively) or plasmacytoid DC (pDC) (Fig. 6a). Cross-presenting cDC1 and pDC are particularly interesting and differ in specific immune functions, which is associated with subset specific gene expression repertoires. TFs are at the top of the hierarchy of gene expression networks driving cell identity and function and thus there is a particular interest in TFs in DC. We perform Omni-ATAC-seq experiments of cDC1 and pDC subsets and employ HINT-ATAC to detect footprints within ATAC-seq peaks for each of these two cell types. Next, we estimate changes in binding activity for 579 TFs with a motif in JASPAR [39]. Cell-specific TF activity is evaluated by measuring the depth of footprints and the total number of reads in flanking regions (see the “Method” section). Higher and lower differences in activity suggest that the TF shows stronger binding in pDC and cDC1 cells, respectively. We also evaluate motifs supported by Wellington footprints or motifs inside ATAC-seq peaks 4. We observe a number of TFs with statistically significant difference in activity between cDC1 and pDC (Fig 6b; *p* value <0.05; *t* test). We further filter TFs by only considering those with an absolute log2 fold change in gene expression higher than 0.5.

Interestingly, the most prominent cDC1 and pDC specific factors, identified by HINT-ATAC, represent two TFs shown to be important in these DC subsets: Batf3 [40] and Tcf4 [41]. Other known DC factors identified by HINT-ATAC include Zeb2, which was shown recently to be crucial in pDC differentiation [42], and Spi1 (also referred as PU1 or SFPI1), which is a master regulator of DC differentiation and mostly active in cDC [38]. The peak-based approach only detects significant activity changes for Batf3, while Wellington only predicted significant changes for Batf3 and Tcf4.

The higher precision of HINT-ATAC is also reflected in the higher AUPR for Batf3 (Fig. 6c), as supported by Batf3 ChIP-seq data in cDC1 from [40]. The average cleavage profiles for Batf3 and Tcf4 (Fig. 6d) and other selected TFs (Additional file 1: Figure S25) further exemplify this. The naive peak-based method is less specific and predicts five times more binding sites than footprinting methods. There is only a partial overlap between Wellington and HINT-ATAC footprints, i.e., 52.18% of footprints predicted by Welligton were also predicted by HINT-ATAC (footprints sharing at least 50% of bases). Notably, average cleavage profiles from Wellington had less signals in flanking regions than HINT-ATAC (Fig. 6d and Additional file 1: Figure S25). This is possibly due to Wellington’s inability to define exact cleavage positions of the Tn5 enzyme. At individual regions, HINT-ATAC footprints are also more specific than other methods in detecting Batf3 binding sites, as exemplified in regions close to the DC relevant genes Irf8 [40] and Flt3 [38] (Fig. 6e).

## Discussion

We demonstrate that the use of position dependency models is crucial for correction of cleavage bias for ATAC-seq and also improves correction for DNase-seq data. As shown in the subsampling experiments, *k*-mer-based estimates are less reliable than the PDMs for libraries with lower sequencing depth, as an indication of overfitting. Dependencies learned by PDMs for both Tn5 and DNase-I are mostly based on adjacent positions. For Tn5, dependencies were detected between the middle of the Tn5 motif and positions 2 bps away. This might indicate a complex dependency of nucleotide recognition by the Tn5 dimer. These higher order dependencies might be associated with DNA shape, as recently demonstrated for DNase-I [29]. Previously, [11] reported difficulties in detecting footprints around motifs associated with regulatory variants in ATAC-seq data. We observed that PDM-based bias correction improves footprint profiles by increasing the footprint shapes for the majority of these motifs (Additional file 1: Figure S26), as an example of the caveats of ignoring cleavage bias.

There is growing attention in the field on cleavage biases present in sequencing protocols using cleavage/digestion enzymes [23, 43]. For example, the “digestion bias” of nucleases has been shown to induce artifacts in nascent RNA-seq protocols [43]. The same enzyme is used in ChIP-seq variants (ChIP-exo [44], ChIP-nexus [45]) and is likely to influence the detection of ChIP-exo footprints. PDMs represent a flexible framework for cleavage bias correction, which is likely to improve downstream analysis of any of these protocols.

The strand-specific cleavage patterns around transcription factor binding sites represent another overlooked aspect of ATAC-seq. Decomposition of DNA fragments by nucleosome number shows intricate strand specific cleavage patterns relative to TF binding. We demonstrate here that the strand bias of ATAC-seq protocols arises from the preference to particular cleavage events. Another evidence of the importance of neighboring proteins to Tn5 cleavage is the fact that the relative predictive power of ATAC-seq in relation to DNase-seq varies for particular TF families. This includes TFs from the bZIP and helix-loop-helix families, which bind as dimers and have large structures. These structural properties are likely to impair access of Tn5 to neighboring DNA regions.

Footprints predicted in Omni-ATAC obtained better performance than standard and fast-ATAC protocols. Omni-ATAC libraries have higher fraction of fragments associated to mono and di-nucleosomes than standard or fast-ATAC protocols. This suggests that improvements introduced in Omni-ATAC protocol enrich for mono and bi nucleosome fragments, leaving more attenuated strand bias profiles in 1N and +2N reads than standard or fast-ATAC-seq. While there is a perception of the field that a large number of reads are necessary for footprint predictions, libraries with moderate number of reads (50 millions) are among the best for ATAC-seq. On the other hand, the quality of the library, as indicated by the fraction of reads within peaks (FRIP), impacts the predictive power of footprints.

Finally, we show how footprints can be used to find TFs associated to DC subset specification. HINT-ATAC has the highest predictive power and identifies four TFs already associated with DC specification. Other predicted TFs with unknown (Zbtb18) or poorly understood (Jdp2) functions in DC development represent interesting candidates for future functional studies. A similar approach was used to identify TFs associated with regulation of beta cells in fasting vs. normal diets with DNase-seq [13]. This study considered all motifs inside DNase-seq peaks. As shown in our analysis, this simple strategy has lower power in detection of cell-specific TFs given the inclusion of a larger number of false positive binding sites.

## Conclusions

We present here the first computational footprinting method tailored to the ATAC-seq protocol. HINT-ATAC corrects the cleavage bias of the Tn5 enzyme with a position dependency model and explores strand-specific bias, which is dictated by the local nucleosome architecture, to detect footprints. HINT-ATAC predictions outperform competing methods and have similar accuracy when applied to either Omni-ATAC-seq or DNase-seq protocols. This indicates that improvements in protocols and our computational approach make ATAC-seq a competitive alternative to DNase-seq for identifying TF binding sites, even for experiments based on moderate number of reads (∼ 50 millions) and low number of cells (∼ 25.000).

## Method

### HINT-ATAC

HINT is a computational framework for detection of footprints from open chromatin data [7]. It works in two major steps: first, genomic cleavage signals are generated from raw sequencing libraries after filtering reads by fragment size, correction of cleavage bias, and signal normalization. Next, cleavage signals are given as input to a HMM, which segments the signal and finds the location of footprints. HINT-ATAC extends HINT[7] by the proposal of a generalized framework for cleavage event counting and bias correction. This new framework allows cleavage events to be displaced from the read start and is based on a probability distribution assuming dependency between nucleotide positions. This allows to consider bias spanning larger genomic areas. HINT-ATAC also extends HINT by the use of strand-specific and fragment-size decomposition of cleavage signals as input. Finally, HINT-ATAC includes a novel semi-supervised training procedure, which uses a single TF ChIP-seq dataset for training. In contrast, the first version of HINT required the manual specification of the topology or data annotation for training [7]. Here, we will describe these novel aspects of HINT-ATAC.

### Cleavage event counting and correction of sequence bias

For a given open chromatin library and a reference genome sequence *G* with length *N*^5^, the first step is to generate the strand-specific genomic signals by counting the cleavage events on the positive or negative strands. HINT-ATAC considers the first position of aligned DNase-seq reads as the cleavage event as usual in the literature [7, 19]. For ATAC-seq, the middle of the Tn5 cleavage event is the fifth base after the fragment start (see Fig. 1a). Given an ATAC-seq read aligned with start position *i*, HINT-ATAC considers the position *i*+4 as a cleavage event for forward reads and *i*−5 for reverse reads^6^. This is equivalent to shifting positions of ATAC-seq reads as originally proposed in [5].

More formally, the strand-specific signals are defined by the vectors:

1 $$  \begin{aligned} \mathbf{y}^{+} = \left\langle {y}_{1}^{+}, \cdots, {y}_{i}^{+}, \cdots, {y}_{N}^{+} \right\rangle \, \, \, \\ \mathbf{y}^{-} = \left\langle {y}_{1}^{-}, \cdots, {y}_{i}^{-}, \cdots, {y}_{N}^{-} \right\rangle \,, \end{aligned}  $$

where ${y}^{+}_{i} \left ({y}^{-}_{i} \right)$ indicates the number of cleavage events at a given genomic position *i* at positive (negative) strands. In the case of paired end reads, it is possible to obtain the fragment length $\mathscr {l}$, by considering the differences between left-most and right-most alignment positions of the read pair. HINT-ATAC also counts cleavage events by only considering reads in a particular fragment size range ($\mathscr {m} < \mathscr {l} \leq \mathscr {n}$), which we denote $\mathbf {y}^{+}(\mathscr {m},\mathscr {n})$. See below for definitions of intervals. In the following, we only consider the positive strand for simplicity.

Next, we correct the cleavage event profiles by sequence-specific cleavage bias considering the word *w*[*i*] with size *k* around genomic position *i*. For a given genome sequence *G*, this is defined as *w*[*i*]=*G*[*i*−⌊*k*/2⌋,*i*+⌈*k*/2⌉−1].

For an arbitrary word *w*, the bias is defined as

2 $$  b(w) = \frac{p(w|obs)}{p(w|exp)},  $$

where *p*(*w*|obs) is the probability of *k*-mer *w* around the position of a cleavage event and *p*(*w*|*exp*) is the probability of finding word *w* in the genome. For a position *i* in the genome, the bias-corrected signal is obtained as [19]:

3 $$  {x}_{i} = (y_{i}+1)/\left(\hat{y}_{i} \cdot \hat{b}(w[i])+1 \right) \,,  $$

where $\hat {y}_{i} =\frac {1}{50} {\sum \nolimits }_{j=i-25}^{i+24} y_{j}$ represents the average number of cleavage events around position *i* and $\hat {b}(w[\!i]\!)= b(w[\!i]\!)/{\sum \nolimits }_{j=i-25}^{i+24} b(w[\!j]\!)$ is the bias of *w*[ *i*] normalized by the bias of surrounding genomic regions.

We describe below three distinct approaches to calculate cleavage bias estimates *b*(*w*): (i) the *k*-mer-based approach, which is widely used in the DNase-seq literature, (ii) a PWM-based approach, which is standard for modeling DNA-protein interactions, and (iii) our novel approach using PDMs.

#### k-mer-based estimation

The most common approach for bias estimation is to use the frequency of *k*-mers to estimate the probability *p*(*w*) [8, 11, 19, 20, 30]. Let *W*^*obs*^ be a multiset with all words with length *k* around individual cleavage events of an open chromatin library and *freq*(*w*|*obs*) be the frequency of a word *w* in *W*^*obs*^. Then, the probability is estimated as

4 $$  p(w|obs)=\frac{freq(w|obs)}{|W^{obs}|},  $$

where |*W*^*obs*^| denotes the total number of *k*-mer occurences $\left | W^{obs} \right | = {\sum \nolimits }_{w} freq(w|obs)$. Similarly, *p*(*w*|*exp*) is estimated on the background multiset (*W*^*exp*^). These estimates are plugged in Eq. 2 to obtain the final cleavage bias estimates. Equation 4 is equivalent to estimating a multinomial distribution of *k*-mers in multisets *W*^*obs*^ and *W*^*exp*^. Estimates are prone to overfitting for large *k* or low number of reads.

As standard in the field [8, 19], we define background regions as all accessible genomic regions, i.e., ATAC-seq or DNase-seq peaks of the corresponding library. An exception are naked DNA experiments, where the complete genome sequence is considered.

#### PWM-based estimation

An alternative approach, which is standard for transcription factor binding models [46], is the use of models assuming independence between positions of *w*. That is,

5 $$  p(w|obs) = \prod\limits_{j=1}^{k} p(w_{j}=b|obs),  $$

where *w*_*j*_ is the *j*th position at word *w* and *b*∈{*A*,*C*,*G*,*T*} a genomic base. We define *freq*_*j*_(*b*|*obs*) as the frequency of base *b* to occur in position *j* in all words in multiset *W*^*obs*^. Then, we can estimate the probability as

6 $$  p(w_{j}=b|obs)=\frac{freq_{j}(b|obs)}{|W^{obs}|}.  $$

Estimates for *p*(*w*|*exp*) are calculated similarly from the background multiset *W*^*exp*^.

#### PDM-based estimation

A main disadvantage of PWM-based estimation is that it assumes statistical independence between different positions in *w*. An alternative between models considering all dependencies (k-mer approach) and no dependencies (PWM approach) is provided by a position dependency model (PDM). PDMs consider dependencies between particular pairs of positions *j* and *l* up to a particular distance *d*, i.e., *d*≥|*l*−*j*| and *l*<*j*. We propose here the use of a special class of PDMs (SLIM models) [26] to estimate the probability of *w*, that is

7 $$ {\begin{aligned}  p(w) = \prod\limits_{j=1}^{k} \left(p(C_{j}=0) \cdot p(w_{j}) + p(C_{j}=1) \sum_{\substack{l\in [1,k]\\j-l \leq d}} p(R_{lj}) \cdot p(w_{j}|w_{l}) \right) \,, \end{aligned}}  $$

where *p*(*C*_*j*_) is the prior probability that the distribution at position *j* should be modeled conditional on other positions; *p*(*R*_*lj*_) is the prior probability that position *j* should be conditional on position *l*; *p*(*w*_*j*_) is the probability of a base to appear in position *j* and *p*(*w*_*j*_|*w*_*l*_) is the probability of base in position *j* conditional on the base from position *l*. It is worth noting that the PWM-based estimation is a special case of SLIM models with *p*(*C*_*i*_=0)=1 for all positions.

For a given multiset *W*, estimates *p*(*w*_*j*_) follow Eq. 6. Conditional estimates *p*(*w*_*j*_|*w*_*l*_) can be derived analogously using frequencies of bi-nucleotides found in positions *j* and *l* in a multiset. The missing estimates *p*(*C*_*j*_) and *p*(*R*_*lj*_) are obtained with a discriminative maximum supervised posterior principle, which uses random sequences as negative models, see [26] for more details. These methods are applied on multisets *W*^*obs*^ and *W*^*exp*^ to obtain *p*(*w*|*obs*) and *p*(*w*|*exp*), which are then used as final bias estimates with Eq. 2. In our experiments, we only consider dependencies such that *d*<6.

### Strand and nucleosome number decomposition and signal post-processing

For paired-end ATAC-seq libraries, we also filter signals by only considering cleavage events from paired-ends with a particular size range $\left (\mathbf {y}^{+}(\mathscr {m},\mathscr {n}) \right)$. We define distinct fragment size intervals with contain distinct number of nucleosomes by estimating local minima between modes of the fragment size distribution of the ATAC-seq library used for model training (Additional file 1: Figure S11). For standard ATAC-seq, the first interval (0,145] represents nucleosome-free reads (Nfr), the interval (145,307] represents one nucleosome reads (1N), the interval (145,*∞*] represents one or more nucleosome reads (+1N) and the interval (307,*∞*] represents two or more nucleosomes (+2N). We then evaluate distinct strategies with combinations of these signals: all reads (All), signal from nucleosome-free reads (Nfr), signals from nucleosome-free reads and signals from reads with one or more nucleosomes (Nfr & +1N), and signals from nucleosome free, signals from one nucleosome, and signals from one or more nucleosomes (Nfr & 1N & +2N).

For each decomposition strategy, we apply the cleavage bias correction, a within-signal normalization by averaging non-zero counts inside bins and a between-signal normalization by applying a logistic function. To estimate the slope of the signals, we apply a Savitzky–Golay smoothing filter by fitting the data into a second order polynomial and performing a convolution (based on a specific window length) with a vector containing Savitzky–Golay coefficients. Normalization steps are described in details in [7].

For example, in the case of All cleavage events with strand specific signals, we have a signal and a slope value for either positive and negative strands, that is:

8 $$  X = \left\{ x_{\text{norm}}^{+}, x_{\text{slope}}^{+}, x_{\text{norm}}^{-}, x_{\text{slope}}^{-} \right\}.  $$

### HMM training and decoding

We take the previously described multivariate cleavage signals *X* as input for the HMM model. The HMM contains a multivariate normal density function with full covariance matrix as emission probability for each state. The dimension depends on the decomposition strategy and varies from 2 (non-strand specific) to 12 dimensions (strand-specific models considering 3 distinct fragment size intervals). For a given TF, we obtain regions with ChIP-seq peaks and a motif predicted binding site (MPBS) as described in the “Evaluation of footprinting prediction” section. We then estimate the average ATAC-seq profile for the region ± 500 bp to the motif center and annotate the center with the label FOOTPRINT. Next, we use a fully connected HMM with *S* states and select one state to represent the FOOTPRINT. We use a semi-supervised algorithm to train the HMM [47]. This algorithm learns the parameters of the HMM in a supervised manner for the FOOTPRINT state and using the Baum-Welch algorithm for all other *S*−1 states (see Additional file 1: HMM Training). The initial model parameters are obtained after execution of a M-step with random posterior distributions with the exception of the FOOTPRINT state, which has the posterior distribution defined by the labels.

To detect footprints in a novel sequencing library, we use the Viterbi algorithm [49] to find the most probable sequence. We consider positions annotated with the FOOTPRINT state to be the active TFBS. This approach has clear advantages over previous HMM-based footprinting methods [7, 8, 50], which require the manual specification of a HMM topology and manual annotation of training data to estimate models. We have evaluated the use of different ChIP-seq datasets of several factors for GM12878 cells for training (CTCF, EGR1, SP1, USF2 and ZNF143). We observed no statistical differences between the models. Thus in order to simplify experimental design, we have arbitrarily selected the model based on EGR1 ChIP-seq in GM12878 cells. The model was employed in all further experiments including ATAC-seq from other cells. EGR1 ChIP-seq was excluded from any evaluation. The number of states *S* can also be varied and will be therefore optimized for each protocol and signal decomposition.

### Cell-specific TF activity

We propose here a simple statistic (activity score-ACT) to measure the strength of TF binding in a particular biological condition. First, we identify all binding sites of a particular TF overlapping with footprints $F=\left \{ \left (f^{l}_{1},f^{r}_{1}),\ldots,(f^{l}_{n},f^{r}_{n} \right) \right \}$, where *f*^*l*^ and *f*^*r*^ represent the leftmost and rightmost genomic positions of the binding site. The activity score of a TF is defined as

9 $$ {\begin{aligned}  ACT(TF) \,=\, \frac{1}{|F|} \sum\limits_{\left(f^{l},f^{r}\right) \in F}\left(\text{} \frac{1}{2}\sum\limits_{j=f^{l}-e-1}^{f_{l}-1} x_{j} \,+\, \frac{1}{2} \sum\limits_{j=f^{r}+1}^{f^{r}+e+1} x_{j} \,-\, \sum\limits_{j=f^{l}}^{f^{r}} x_{j}\right) \,+\, \left(\sum\limits_{j=f^{l}-100}^{f^{r}+100} x_{j}\right), \end{aligned}}  $$

where *e* is the length of the binding site and *x*_*j*_ is the cleavage event signal (after bias correction) at genomic position *j*.

The activity score can be seen as a combination of the protection score [8], which measures the difference in cleavage events between the footprint and flanking regions (left term), and the openness score [51], which simply measures the number of cleavage events around the binding site (right term).

Here, we are interested in identifying TFs with change in activity between two conditions. This is given by the difference in ACT scores between two biological conditions, this is:

10 $$  \Delta ACT(TF) = \frac{ACT_{2}(TF)}{\omega_{2}} - \frac{ACT_{1}(TF)}{\omega_{1}},  $$

where *ω*_1_ and *ω*_2_ are normalization factors based on median-of-ratios [52]. These factors correct for differences of sequencing depths of libraries of the two conditions. Here, the set *F* corresponding to binding sites supported by footprints in at least one of the conditions. We use this score to rank TFs with a known motif, where highest *ΔACT*(*TF*) indicate TFs with specific binding in condition 2.

### Materials and experimental design

#### Low level analysis of DNase-seq and ATAC-seq libraries

We used ATAC-seq data of cell lines GM12878 and K562 from [5, 6, 28, 53] and single-cell ATAC-seq data from cell lines GM12878, K562, H1-ESC [33], mouse blood cells from [54], and DNase-seq data of GM12878, K562, and H1-ESC from [55]. We also used naked DNA ATAC-seq data from [21] and naked DNA DNase-seq data from [30] for estimation of cleavage bias.

First, adapter sequences were trimmed from FastQ files using Trim Galore [56] with the following settings (-q 30 –paired –trim1). Reads were mapped to the reference genome using Bowtie2 [57] with the following parameters (-X2000 –no-mixed –no-discordant) allowing fragments of up to 2 kb to align. Duplicates were removed and reads were filtered for alignment quality of >Q30 using samtools [58]. Next, MACS2 [59] was used to call ATAC-seq or DNase-seq peaks with the following parameters (–nomodel –nolambda –keep-dup auto –call-summits). The overlapping peaks were merged and then filtered for q-value >10. The same preprocessing was applied to naked DNA ATAC-seq and DNase-seq except for peak calling. All organism-specific data are based on human genome build 37 (hg19) and mm10. Reads mapping to the mitochondria, unmapped contigs and chromosome Y were removed from all subsequent analyses. Concerning ATAC-seq experiments performed on single cells, we combined all sequence libraries to consider them as a bulk experiment. See Additional file 2 for complete list of libraries and quality statistics.

#### ATAC-seq on dendritic cell specification

Dendritic cells (DC) are professional antigen presenting cells that comprise different subsets: classical DC type1 and type2 (cDC1 and cDC2, respectively) and plasmacytoid DC (pDC). In this study cDC1 or pDC were obtained in a two-step in vitro culture system according to [37]. Briefly, mouse bone marrow cells were first amplified with a specific cytokine cocktail and then induced to differentiate into DC with Flt3 ligand. cDC1 are CD11c+ CD11b+ XCR1+ and pDC are CD11c+ CD11b- B220+ and thus cDC1 and pDC subsets were purified by FACS sorting and subjected to Omin-ATAC-seq analysis. Omni-ATAC-seq was performed according to [6] with minor modifications. Prior to transposition dead cells were removed by centrifugation (800 rpm, 4 min, 4 °C). The transposition reaction was with 7.5 *μ*L Tagment DNA Enzyme 1 (TDE1) for 60 ‘min at 37 °C. Pre-amplification was with NEBNext Ultra II Q5 Master Mix and Nextera PCR Primers (5 cycles). Quantitative PCR amplification was with NEBNext Ultra II Q5 Master Mix, Nextera PCR Primer and SYBR Gold to determine the number of additional cycles. PCR amplification of additional cycles was as for pre-amplification. PCR fragments were purified with Qiagen MinElute PCR Purification Kit and library concentration and quality were determined by Agilent High Sensitive DNA Kit and Bioanalyzer, respectively. ATAC-seq libraries were sequenced with a Illumina NextSeq 500 Platform with 75 bps paired-end reads in duplicates. Trimming, alignment (to mouse genome mm9), and peak calling were performed as for other ATAC-seq libraries. Replicate libraries were merged previously to footprinting.

#### Evaluation of footprinting prediction

ChIP-seq of TFs and motif-predicted binding sites (MPBSs) were used as ground truth to evaluate the footprinting prediction in this work following [8, 27]. We used here peaks from 124 TF ChIP-seq datasets provided by the ENCODE analysis working group on cell lines K562 (60), H1-ESC (31), and GM12878 (33) [60]. We obtained a PWM for each factor from the Jaspar database [61]. For a few exceptional cases were a motif was not found in Jaspar (5 TFs), we used matrices from Uniprobe [62], or Transfac [63]. See Additional file 1: Table S31 for stastistics of ChIP-seq data and motifs. Next, we used a motif matching tool based on the MOODS C++ library [64] to find MPBS. We determined a bit-score cut-off threshold by applying the dynamic programming approach described by [65] with an FPR of 10^−4^. Then, we created labels by combining MBPSs with ChIP-seq data for every TF. Specifically, MPBSs with ChIP-seq evidence (MPBS located within 100 bp from the ChIP-seq peak summit) are considered true binding sites and MPBSs without ChIP-seq evidence are considered false binding sites. Footprint/MPBS pairs supported by ChIP-seq peaks are considered true positives (TP), while footprints with no ChIP-seq support are considered false positives (FP). TN and FN are defined accordingly. We rank the predictions for all methods by tag count (TC) as this has been shown to be the best method for ranking predictions [8].

To assess the accuracy, we created receiver operating characteristic (ROC) curves and evaluated area under ROC at 100%, 10%, and 1% FPR by using the contingency table (TPs, FPs, TNs, and FNs). We also measured the area under Precision-Recall (auPRC) at 100%, 10%, and 1% recall as these measures are more suitable for data with skew on negative classes [66, 67]. As the relative performance of methods might vary on distinct evaluation measures, we combine these with the approach used in the ENCODE-DREAM Challenge (https://www.synapse.org/#!Synapse:syn6131484/wiki/405275). This score is equal to the sum over all six normalized ranking measures of −*log*(*r*/(*N*+1)) where r is the rank of an algorithm for a specific performance measure (e.g., auROC) and N is the total number of methods. Therefore, best measures should have a high ranking in several of the evaluated measures.

#### Evaluation data sets

We divided our evaluation data in three distinct sets. The first set (training dataset) is composed of 32 TFs ChIP-seq from the GM12878 and different ATAC/DNase-seq data. To evaluate bias correction methods, we used ATAC-seq data from standard protocol based on 50.000 cells [5], Omni protocol [6], Fast protocol [28], and naked DNA [21]. We also used DNase-seq (single hit) of the same cell line from ENCODE and naked DNase-seq from [30] to evaluate the impact of novel bias correction strategies on DNase-seq data. In addition, we included double-hit DNase-seq of GM12878 to evaluate HMM learning strategies. The next data set (test dataset) is based on 148 combinations of TF ChIP-seq and ATAC-seq experiments from H1-ESC and K562 cells. This data set was used to compare HINT-ATAC with other footprinting methods. ATAC-seq data K562 from 50.000 cells from [14] and combined single cell ATAC-seq data from H1-ESC and K562 cells [33] and Omni-ATAC K562 cells from [6]. In above analyses, we excluded the TF ChIP-seq data from EGR1, which was used as label for the model (see Additional file 2 for full description of ATAC/DNase-seq data).

Finally, for the comparative evaluation of ATAC-seq and DNase-seq protocols, we defined a comprehensive data set by combining all TF-ChIP data (124) from previous data sets. This dataset is further enhanced by using single cell ATAC-seq libraries (GM12827, K562). We also annotated transcription factors regarding their structural family in accordance to JASPAR [39]. For statistical power, we only kept families with more than 10 annotated TFs. Concerning the comparison of footprinting performance on enhancer versus promoter regions, we obtained histone based segmentation of the corresponding cells with chromHMM [35] for GM1878 and K562 cells. We combined all states associated with enhancer or promoter regions which were used to split our TFBS prediction sets into two (Additional file 1: Table S31).

Statistical comparison of computational methods regarding TF binding sites was performed with the non-parametric Friedman-Nemenyi hypothesis test. Such a test provides a rank of the methods as well as the statistical significance of the out-performance of a particular method. Comparisons based on non-paired distributions were performed with the Wilcoxon rank sum test. All reported *p* values based on multi-comparison tests were corrected using the Benjamini-Hochberg method.

## Additional files

Additional file 1 Supplementary methods, figures and tables. (PDF 6,084 kb)

Additional file 2 Description of ATAC-seq and DNase-seq data. (XLSX 19 kb)

## Footnotes

1 Query performed using the words “ATAC-seq” and “DNase-seq” at August 16, 2018 considering the number of series and samples deposited within the last year

2 For each cleavage signal, HINT-ATAC also generates a slope signal to help detection of regions with increase/decrease on cleavage sites

3 Given the fact Wellington and DeFCoM do not support multivariate signals, it is only possible to evaluate the Nfr decomposition for these methods.

4 DeFCoM cannot be used in this unbiased analysis as it requires TF ChIP-seq data to train models, which is not given for all 579 TFs deposited in JASPAR.

5 For sake of simplicity, we assume genomes are formed as a single chromosome.

6 An additional base pair is required to define the position of a cleavage event from reads mapped to negative strand (−5 instead of −4), as the right most positions of genomic intervals include an extra base.
