# Supplementary material for: Identification of transcription factor binding sites using ATAC-seq
Source: Genome Biol. 2019 Feb 26;20:45. doi: 10.1186/s13059-019-1642-2 (PMC6391789; doi:10.1186/s13059-019-1642-2)
Supplement: Supplementary file 1 — Supplementary methods, figures and tables. (PDF 6,084 kb) [file 13059_2019_1642_MOESM1_ESM.pdf]

Additional file 1 for Identification of Transcription Factor Binding Sites using ATAC-seq

Zhijian Li[1,2], Marcel H. Schulz[3,4], Thomas Look[2,5], Matthias Begemann[6], Martin Zenke[2,5], and Ivan G. Costa*[1,5]

[1]Institute for Computational Genomics, Joint Research Center for Computational Biomedicine, RWTH Aachen University Medical School, Aachen, Germany.
[2]Department of Cell Biology, Institute of Biomedical Engineering, RWTH Aachen University Medical School, Aachen, Germany.
[3]Cluster of Excellence for Multimodal Computing and Interaction, Saarland Informatics Campus, Saarland University, Saarbrücken, Germany
[4]Computational Biology & Applied Algorithmics, Max Planck Institute for Informatics, Saarbrücken, Germany
[5]Helmholtz Institute for Biomedical Engineering, RWTH Aachen University, Aachen, Germany
[6]Institute of Human Genetics, RWTH Aachen University Medical School, Aachen, Germany

January 16, 2019

*ivan.costa@rwth-aachen.de

1 Supplementary Methods

1.1 HMM Training

A HMM is specified by the following parameters:

1. $S = s_1 s_2 \dots s_N$: a set of hidden states
2. $\pi = (\pi_1 \pi_2 \dots \pi_N)$: initial state probabilities
3. $A = \{a_{ij} | i = 1, \dots, N; j = 1, \dots, N\}$: a state transition probability matrix where $a_{ij} = P(s_{t+1} = j | s_t = i)$ and $\sum_{j=1}^{N} a_{ij} = 1$
4. $B = \{b_n(x_t) | n = 1, \dots, N; t = 1, \dots, T)\}$: emission probability where $b_n(x_t) = P(x_t | s_t = n)$ is a full covariance o-dimensional Gaussian distribution and x_t comes from an observation sequence $X = (x_1, ..., x_t, \dots, x_T)$, where x_t is a o-dimensional vector with signals given as input for the HMM and o varies from 2 to 12 depending of the signal generation strategy.

For simplification, we use $\lambda = (\pi, A, B)$ to indicate the full parameter set of a HMM. Given an observation sequence, i.e., ATAC-seq digestion profiles, we will learn the λ by maximizing the likelihood function $P(X|\lambda)$ using 'semi-supervised' learning .

We start the training with some initial estimate of λ and calculate the forward variable $\alpha_t(i)$ as:

$$\alpha_t(i) = P(x_1, x_2, \dots, x_t, q_t(i) | \lambda) \tag{1}$$

and backward variable $\beta_t(i)$ as:

$$\beta_t(i) = P(x_{t+1}, x_{t+2}, \dots, x_T | q_t(i), \lambda). \tag{2}$$

We can now calculate the probability of being state i at observed sequence position t as:

$$\gamma_t(i) = P(x_t = i | X, \lambda) = \frac{P(x_t = i, X|\lambda)}{P(X|\lambda)} = \frac{\alpha_t(i)\beta_t(i)}{\sum_j^N \alpha_t(i)\beta_t(i)}. \tag{3}$$

and the probability of being state i at t and state j at $t+1$ given as $\xi_t(i,j)$:

$$\xi_t(i,j) = P(q_t(i), q_{t+1}(j) | X, \lambda) = \frac{\alpha_t(i) a_{ij} b_j(t+1) \beta_{t+1}(j)}{P(X|\lambda)} = \frac{\alpha_t(i) a_{ij} b_j(t+1) \beta_{t+1}(j)}{\sum_{i=1}^N \sum_{j=1}^N \alpha_t(i) a_{ij} b_j(t+1) \beta_{t+1}(j)} \tag{4}$$

We have, in our training data, labels corresponding to the footprint state. We use therefore a semi-supervised approach that constraints training for the footprint state to be performed by the normal ML estimates, while other states are estimates with the Baum-welch. This can be obtained by fixing the posterior distribution estimation and follows the framework from (Zhong

2005). Specifically, given an observed sequence $(x_1, x_2, \dots, x_T)$ with an annotated footprint subsequence $(x_m, x_{m+1}, \dots, x_n)$, where $1 \leq n \leq m \leq T$, for $\gamma_t(i)$, the following fixing procedure is used:

$$\gamma_t^{'}(i) = \begin{cases} 0, & \text{if} \quad i \neq s_{fp} \quad \text{and} \quad t < m \quad \text{or} \quad t > n, \quad \text{or} \quad i \neq s_{fp} \quad \text{and} \quad m \leq t \leq n \\ 1, & \text{if} \quad i = s_{fp} \quad \text{and} \quad m \leq t \leq n \\ \dfrac{\gamma_t(i)}{\sum_{i \neq s_{fp}}^{N} \gamma_t(i)}, & \text{otherwise} \end{cases} \tag{5}$$

where s_{fp} is the pre-defined footprint state and $\xi_t^{'}(i,j)$ can be obtained using a similar procedure. In doing so, we correct the expectation estimates by distinguishing the states as footprint state and non-footprint states. Next, the transition probability a_{ij} can be estimated as:

$$\hat{a}_{ij} = \frac{\sum_{t=1}^{T-1} \xi_t^{'}(i,j)}{\sum_{t=1}^{T-1} \sum_{k=1}^{N} \xi_t^{'}(i,k)} \tag{6}$$

the initial state probabilities can be estimated as:

$$\hat{\pi}_i = \sum_{j=1}^{N} \xi_1^{'}(i,j) \tag{7}$$

The observation values of state i are assumed to have a multivariate Gaussian distribution with density function:

$$p(x^i; \mu^i, \Sigma^i) = \frac{1}{(2\pi)^2 |\Sigma^i|^{1/2}} exp(-\frac{1}{2}(x^i - \mu^i)^T (\Sigma^i)^{-1} (x^i - \mu^i)) \tag{8}$$

Finally, the mean μ^i and covariance matrix Σ^i can be estimated as:

$$\hat{\mu}^i = \frac{\sum_{t=1}^{T} \gamma_t^{'}(i) x_t}{\sum_{t=1}^{T} \gamma_t^{'}(i)} \tag{9}$$

$$\hat{\Sigma}^i = \frac{s + \sum_{t=1}^{T} \gamma_t^{'}(i)(x_t - \hat{\mu}^i)(x_t - \hat{\mu}^i)^T}{\sum_{t=1}^{T} \gamma_t^{'}(i)} \tag{10}$$

where s is set to 0.01.

1.2 Competing methods

PIQ (Sherwood et al. 2014) implementation was obtained from `http://piq.csail.mit.edu` and executed with the default parameters, which can be found in the script common.r. Briefly, MPBSs were generated with the script pwmmatch.exact.r. The DNase-seq or ATAC-seq signal was created using the script bam2rdata.r. The footprints were detected with the script pertf.r. DNase2TF (Sung et al. 2014) source code was obtained from `http://sourceforge.net/projects/dnase2tfr/` and executed with the default 4-mer sequence bias correction. The parameters were set to their default values: minw, 6; maxw, 30; z_threshold, 2; FDRs, 10^{-3}. DeFCoM (Quach and Furey 2016) was downloaded from `https://bitbucket.org/bryancquach/`

defcom. Since it is a supervised footprint detection method, we split the dataset into 5 folds and perform training and prediction on each fold, then we merge all predictions for each cell as the final result. Wellington's source code (Piper et al. 2013) was downloaded from http://jpiper.github.com/pyDNase and executed with default parameters. Briefly, we used a footprint FDR cutoff of 30, footprint sizes varying between 6 and 40 with 1 bp steps and shoulder size (flanking regions) of 35 bp.

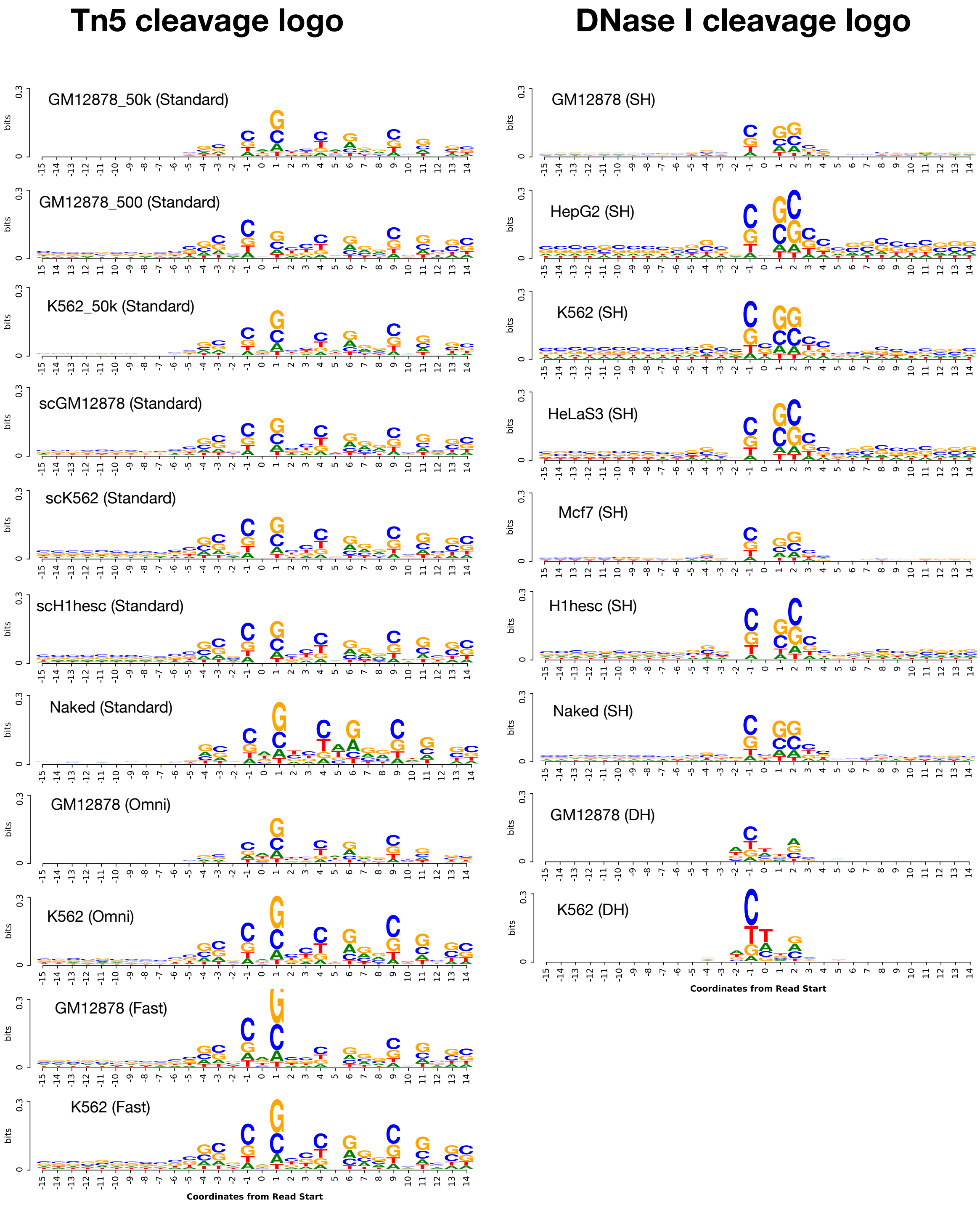

Figure S1: Cleavage logo corresponding to Tn5 and DNase I binding as measured on distinct ATAC-seq (standard, Omni and Fast) and DNase-seq [single hit (SH) and double hit (DH)] protocols and cells. Position 1 corresponds to the start position of the ATAC/DNase-seq read. Libraries from ATAC-seq, DNase-seq SH or DNase-seq DH protocols have small variations of the same motif.

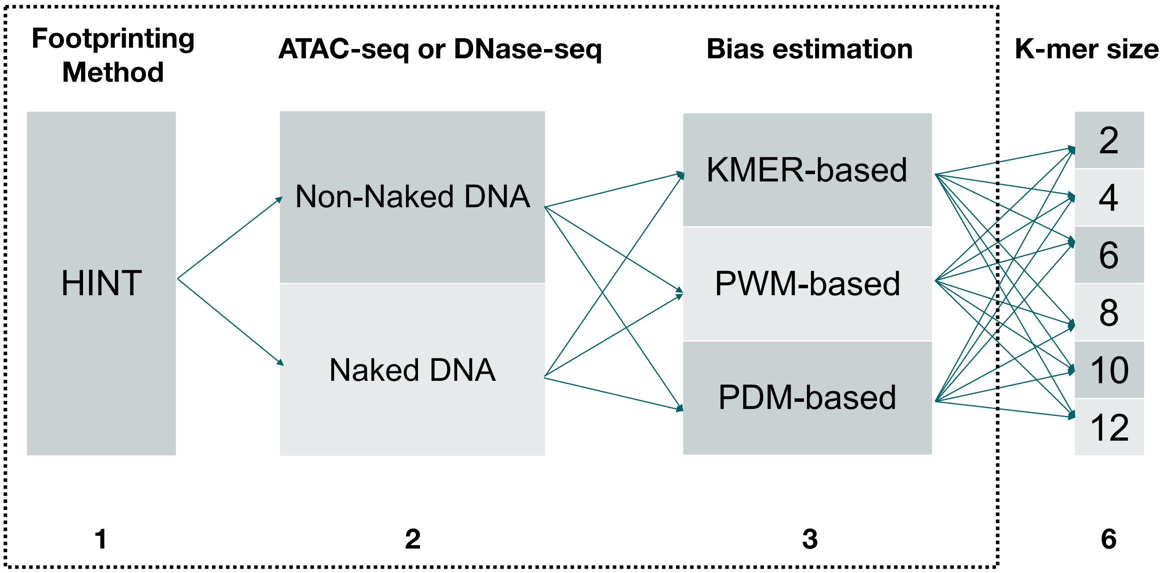

Figure S2: Experimental design for evaluation of bias correction methods on GM12878 cells. We evaluate all 36 methods/parameters depicted above. We first select the best k-mer size for each combination of experiment and bias estimation methods (6 combinations inside the dashboard) and then compare the remaining combinations. The above selection of bias correction methods is independently performed for each ATAC-seq and DNase-seq protocol.

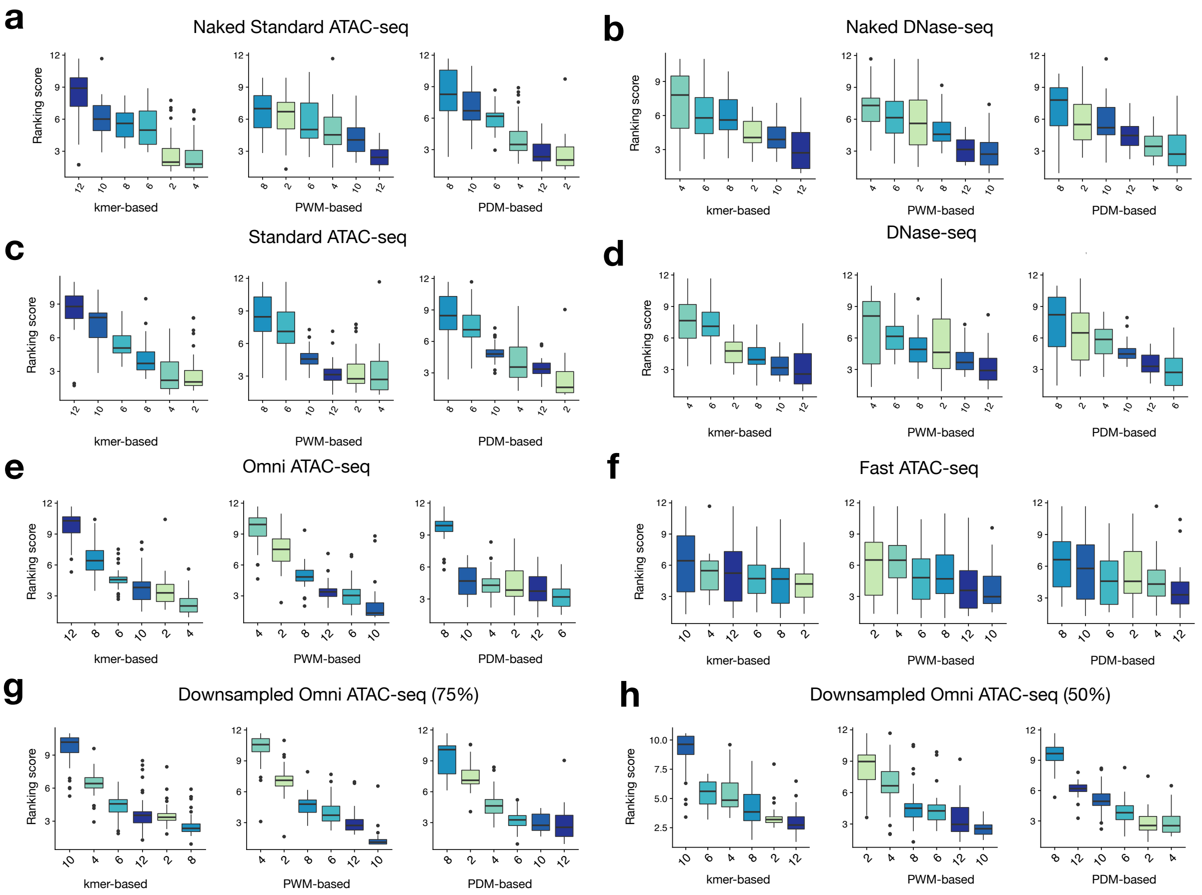

Figure S3: Ranking score contrasting the word size used for each combination of bias correction methods for naked ATAC-seq (**a**), naked DNase-seq (**b**), standard ATAC-seq (**c**), DNase-seq (**d**), Omni ATAC-seq (**e**), Fast ATAC-seq (**f**), Omni ATAC-seq libraries downsampled to have only 75 % (**g**) and 50% (**h**) of reads. All experiments are based on GM12878 cells.

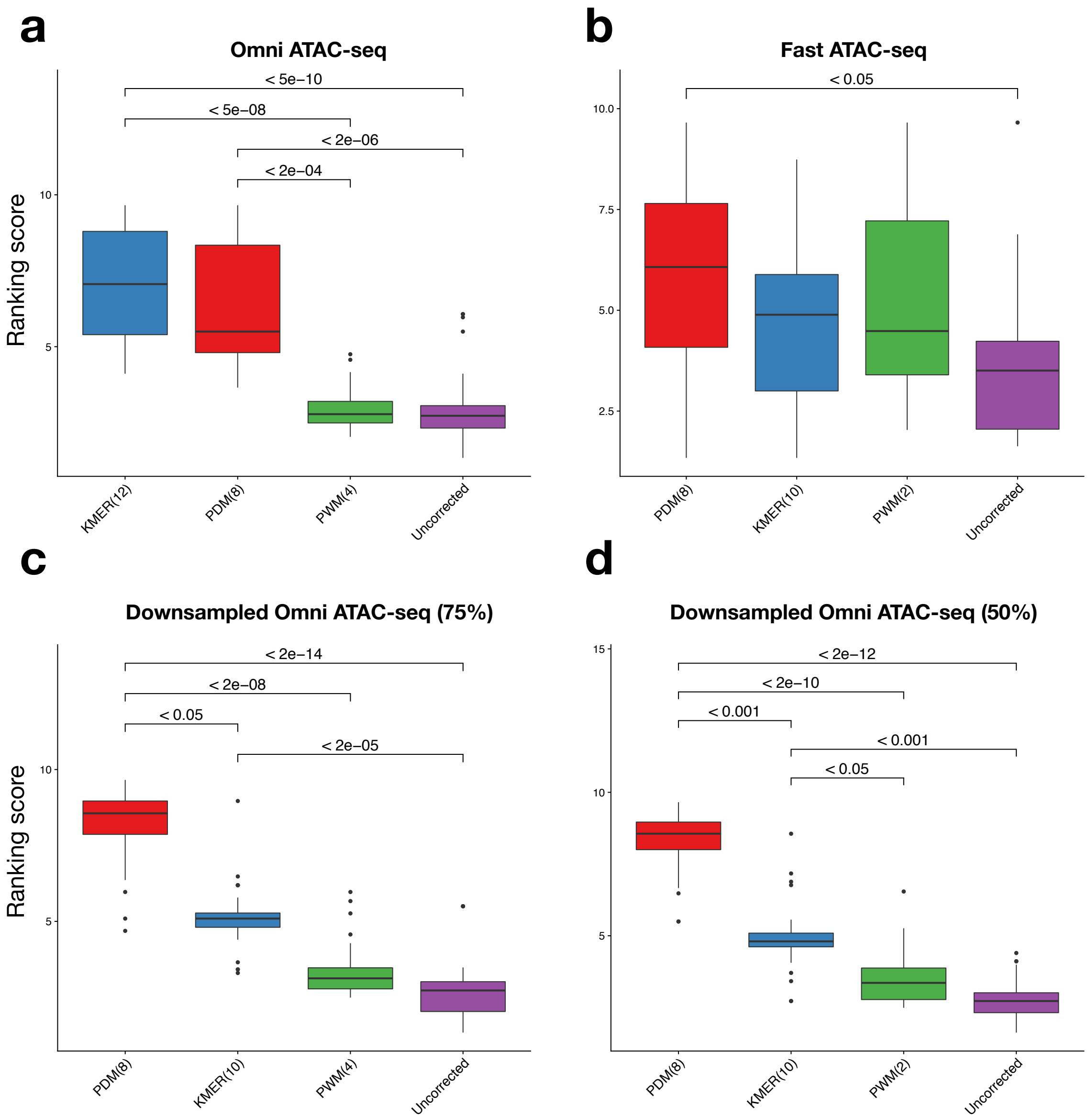

Figure S4: Ranking score contrasting the bias estimation methods for Omni ATAC-seq (**a**), Fast ATAC-seq (**b**), Omni ATAC-seq downsampled to 75 % (**c**) and 50 % (**d**). We selected the best word size (k) for each method according to the comparison results of Fig. S3 (see Tables S1-S12 for statistics test results).

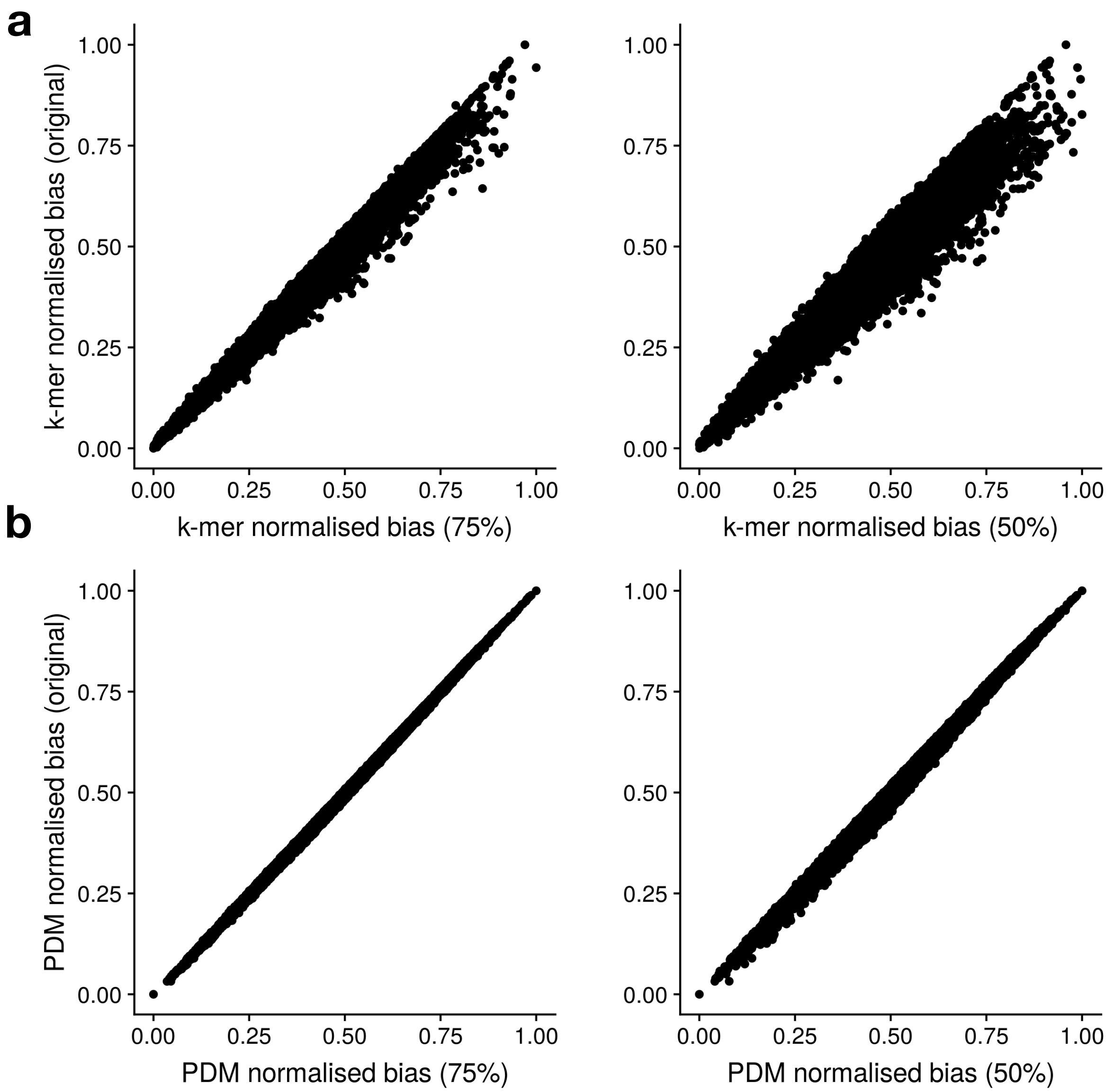

Figure S5: Scatter plot with normalised cleavage bias estimates of k-mer (**a**) and PDM (**b**) models for the original GM12878 Omni-ATAC-seq library (y-axis) vs. downsampled versions with 75% or 50% of reads (x-axis). This Omni ATAC-seq has originally 70 million reads. Bias estimates were normalised as in Lazarovici et al. (2013). We observe higher variance in bias estimates for k-mer based approach than for PDM based approach.

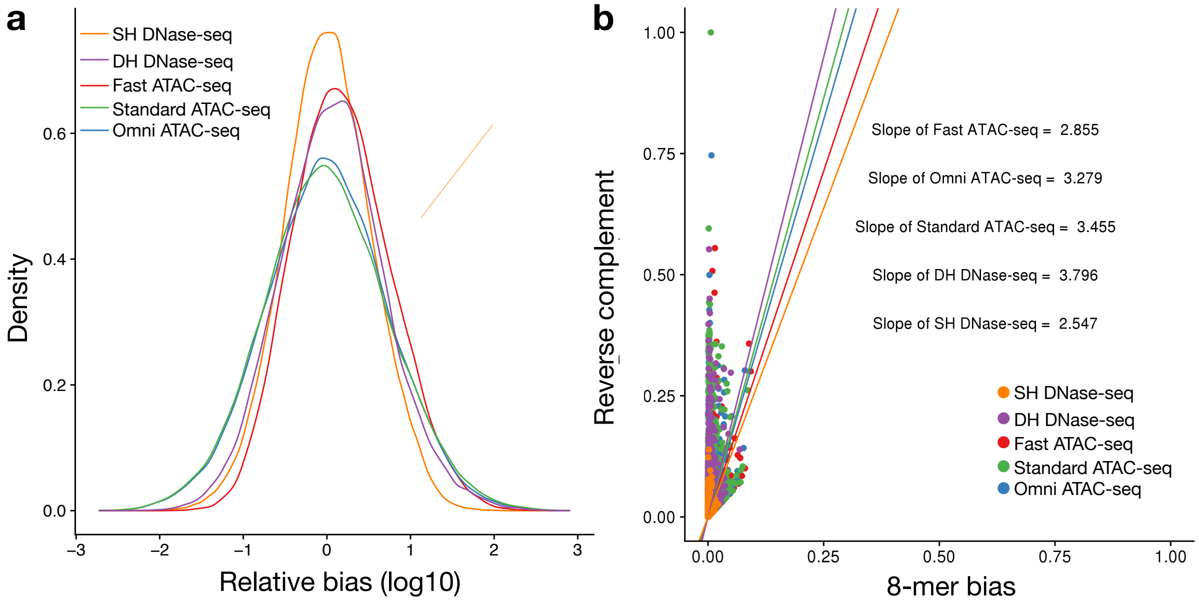

Figure S6: (**a**) Distribution of 8-mers bias estimates from PDM for distinct protocols in GM12878 cells. Relative bias (x-axis) corresponds to log transformed values from Eq. 2 (main manuscript). We observe that DNase-seq estimates are closer to zero than ATAC-seq protocols. This indicates higher relative bias of Tn5 enzyme than DNase-I for particular k-mers. (**b**) Normalised PDM cleavage bias comparing of 8-mers (x-axis) and its reverse complement (y-axis). Palindromic sequences are ignored and only points above the diagonal are show as in Lazarovici et al. (2013). Fitted slopes indicates that estimates are distributed far from the diagonal, which supports the non-palindromic nature of both Tn5 and DNase-I bias estimates.

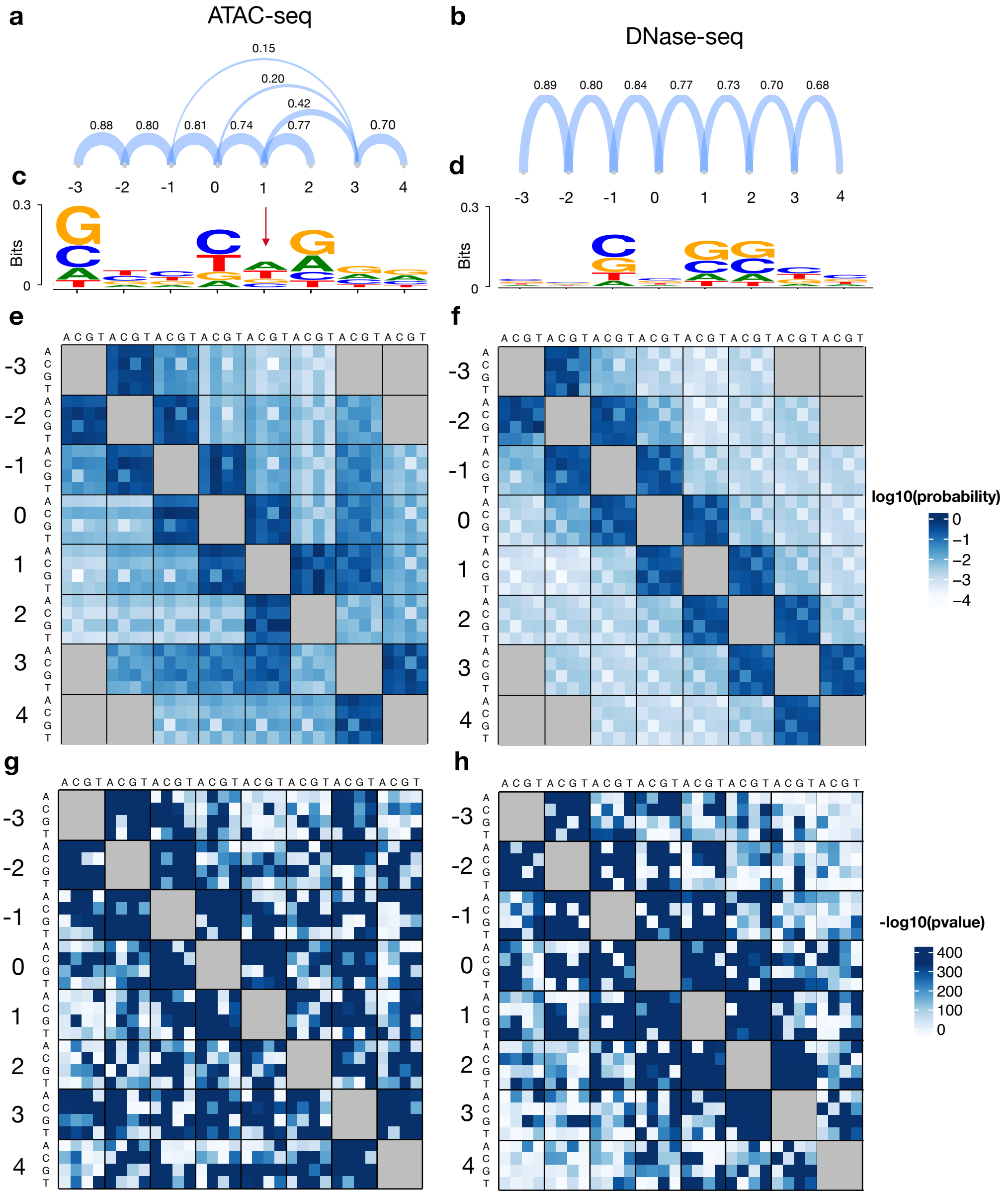

Figure S7: Dependencies considered by PDM model [parameter $p(C_j)$ in Eq. 7 of main text] for standard ATAC-seq (**a**) and DNase-seq (**b**) in GM12878 cells. Position 1 corresponds to the digestion event, i.e. position 5 of a forward ATAC-seq read and first position of a DNase-seq read. Only dependencies with $p > 0.1$ are shown. Logos corresponding to these positions (**c-d**). Interestingly, first order dependencies are relevant for both protocols. However, for ATAC-seq, higher order dependencies are considered among positions -1,0,1 and position 3. These dependencies are reflected in the conditional probabilities considered by PDM [parameter $p(R_{lj})$ in Eq. 7 of main text] for ATAC-seq (**e**) and DNase-seq (**f**). Grey values indicate dependencies not considered by PDM (order higher than 5). Additionally, we performed the statistical test to measure dependencies between pairs of conditions proposed in Lazarovici et al. (2013) (**g-h**). We observed a similar dependency pattern as captured by PDM (**e**, **f**).

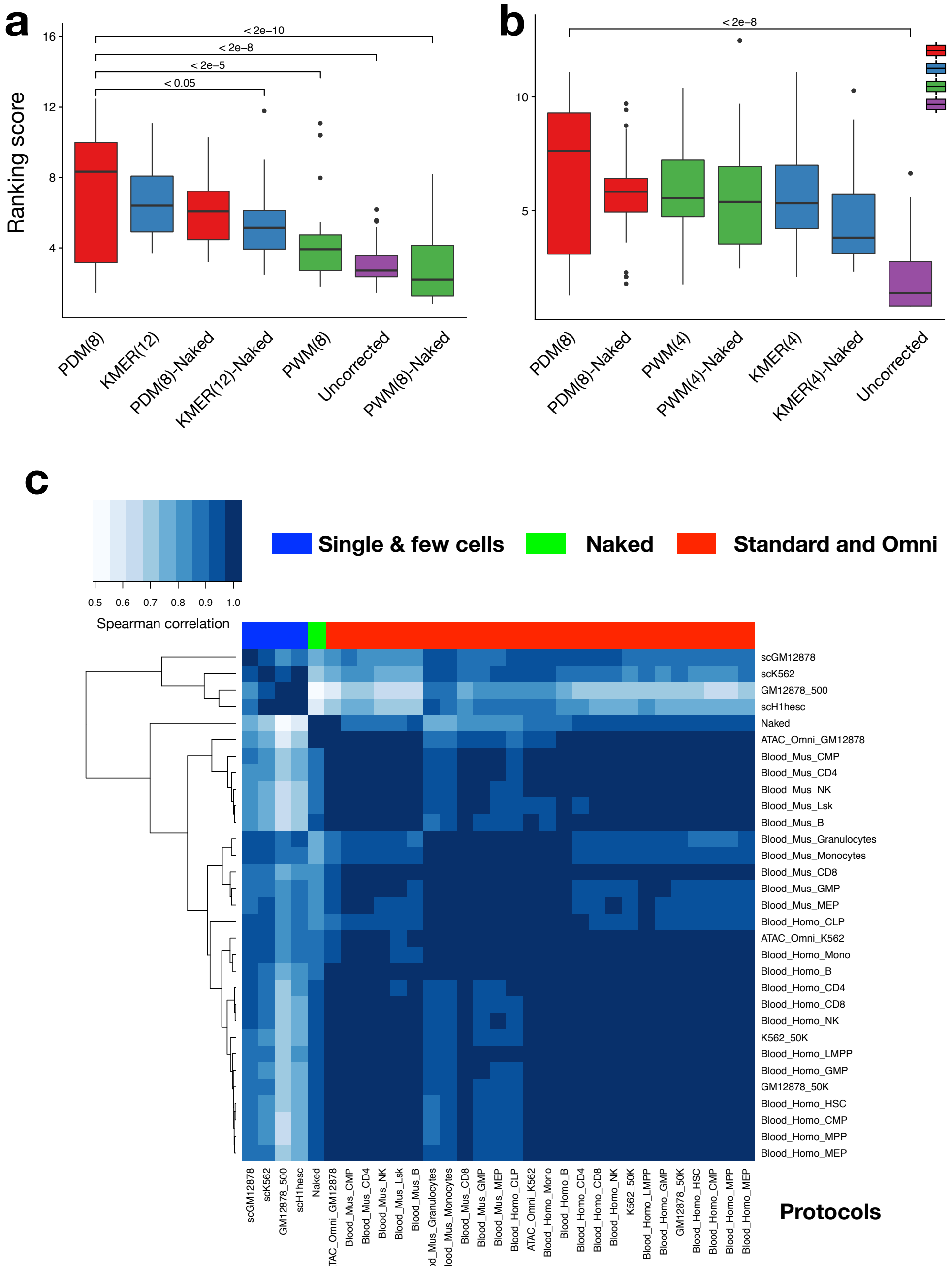

Figure S8: Comparison of bias estimation methods in standard ATAC-seq (**a**) and DNase-seq (**b**) on 32 TF ChIP-seq data sets from GM12878 cells. Bias estimates are either computed in the ATAC-seq libraries themselves or based on ATAC-seq performed in naked DNA (`Naked`). Overall, models based no Naked DNA performed worst (lowest rank) than the corresponding model using bias estimates from the GM12878 ATAC-seq library. (**c**) Hierarchical clustering of ATAC-seq bias estimates using PDM-based approach on several ATAC-seq libraries. Clustering is based on Spearman's rank correlation coefficient and McQuitty criteria between the sequence bias. We observed three clusters: one small cluster with single cell or experiments with few cells, a large cluster with all cells based on bulk cells (standard, Omni or Fast) and an outlier cluster based on a naked DNA experiment. The distinct bias in protocols with few cells can be an effect of over-digestion of chromatin by Tn5 (Buenrostro et al. 2015).

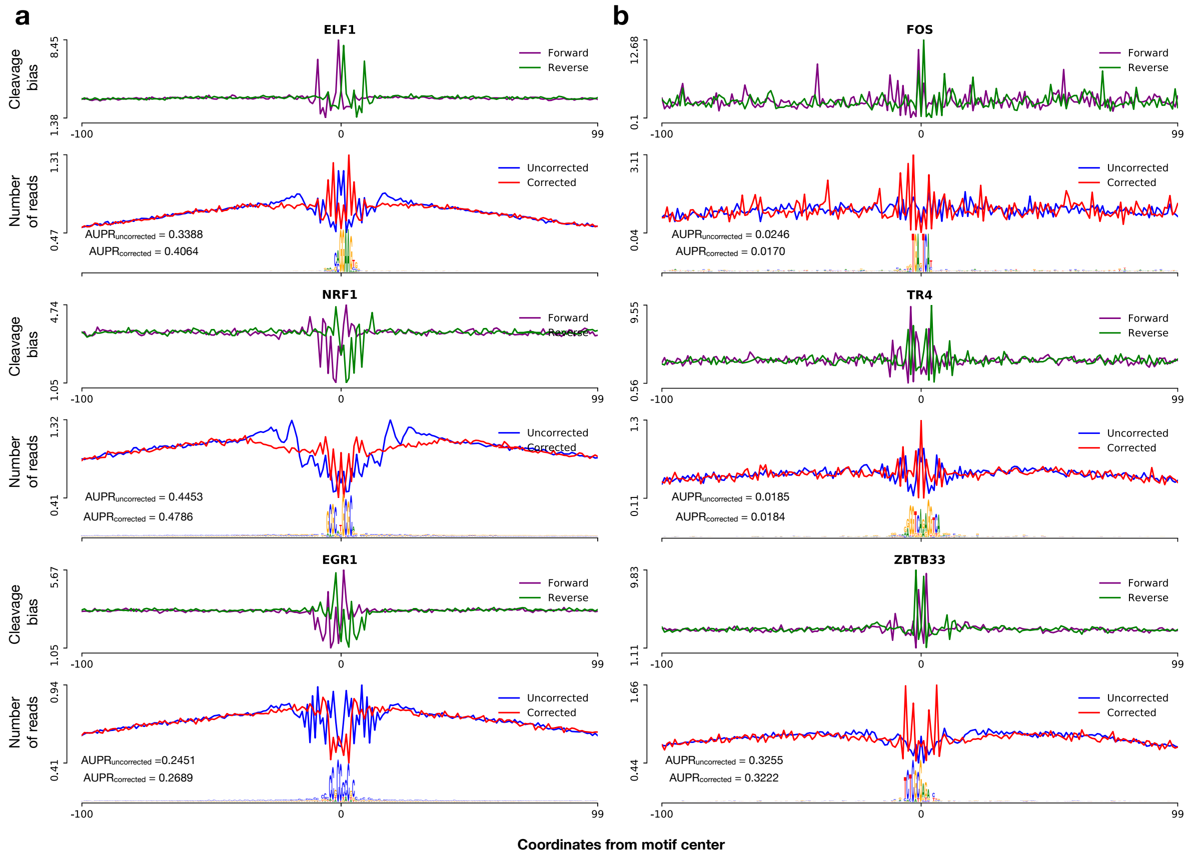

Figure S9: Strand specific cleavage bias with corrected (PDM based) and uncorrected ATAC-seq signal profiles around ChIP-seq supported binding sites of factors with an increase (**a**) and decrease (**b**) in AUPR after PDM based bias correction. All ATAC-seq profiles are based on GM12878 cells.

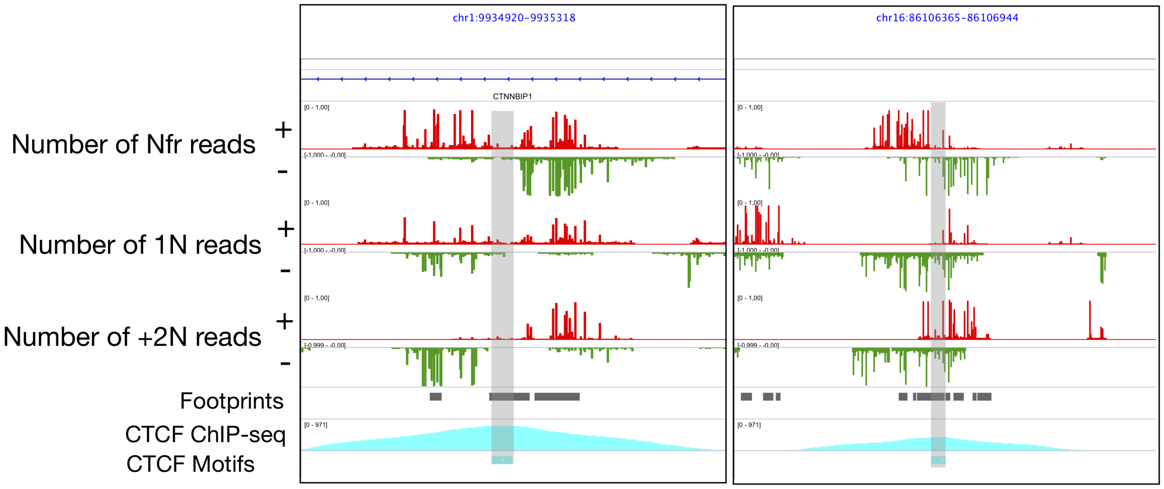

Figure S10: Strand specific Omni-ATAC-seq profiles around genomic regions with CTCF ChIP-seq supported binding sites in GM12878 cells. Figure also includes footprint predicted by HINT-ATAC, CTCF motifs and CTCF ChIP-seq signals. We observe higher number of forward reads (red) in `Nfr` signals left to CTCF, while more reverse reads (green) are seen right of CTCF for `1N` and `+2N` signals.

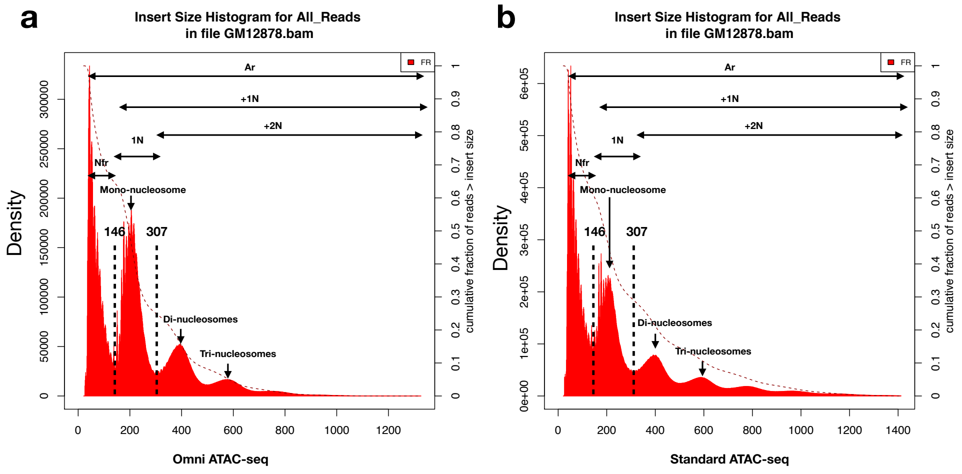

Figure S11: Distribution of fragment sizes for Omni ATAC-seq (**a**) and standard ATAC-seq (**b**) on GM12878. Cutoffs 146, and 307 are determined by searching for local minimum of fragment counts between the first three modes. Reads with size below 146 are considered nucleosome free (`Nfr`), reads between 146 and 307 are considered as mono-nucleosomes (`1N`) and above 146 are more than one nucleosomes(`+1N`), reads larger than 307 are considered to contain two or more nucleosomes (`+2N`). For Fast ATAC-seq, the optimal cutoffs are 122 and 295.

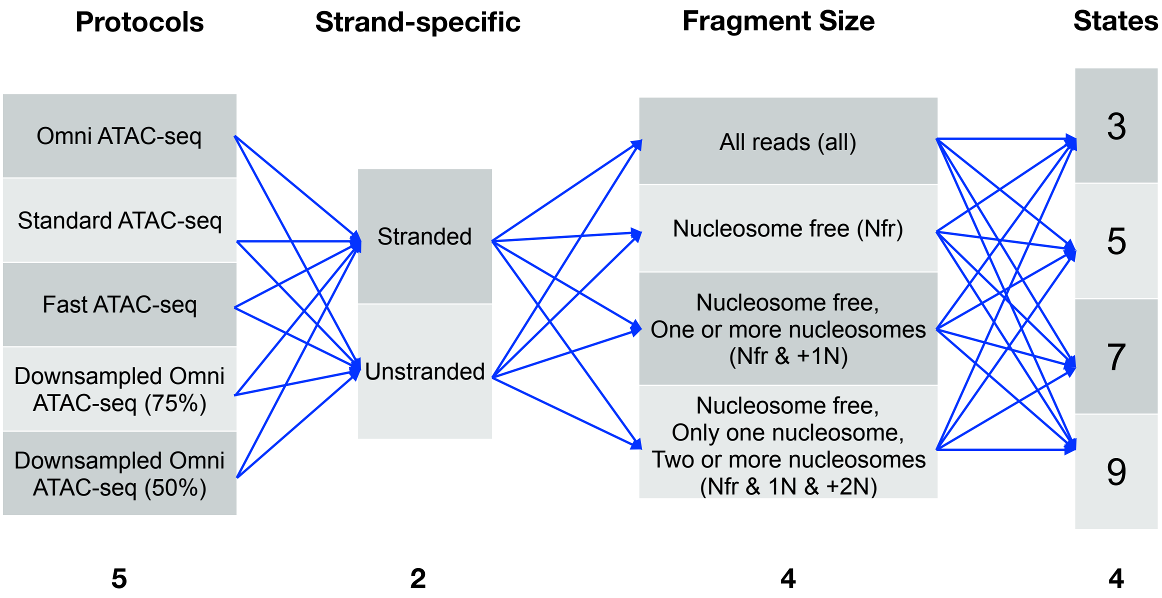

Figure S12: Experimental design for model selection of HINT-ATAC. We evaluate here models for Omni, standard, Fast ATAC-seq protocols and downsampled Omni ATAC-seq (75% and 50%). The models include strand specific and non-strand specific signals, four combinations of nucleosome decompositions and of HMM states.

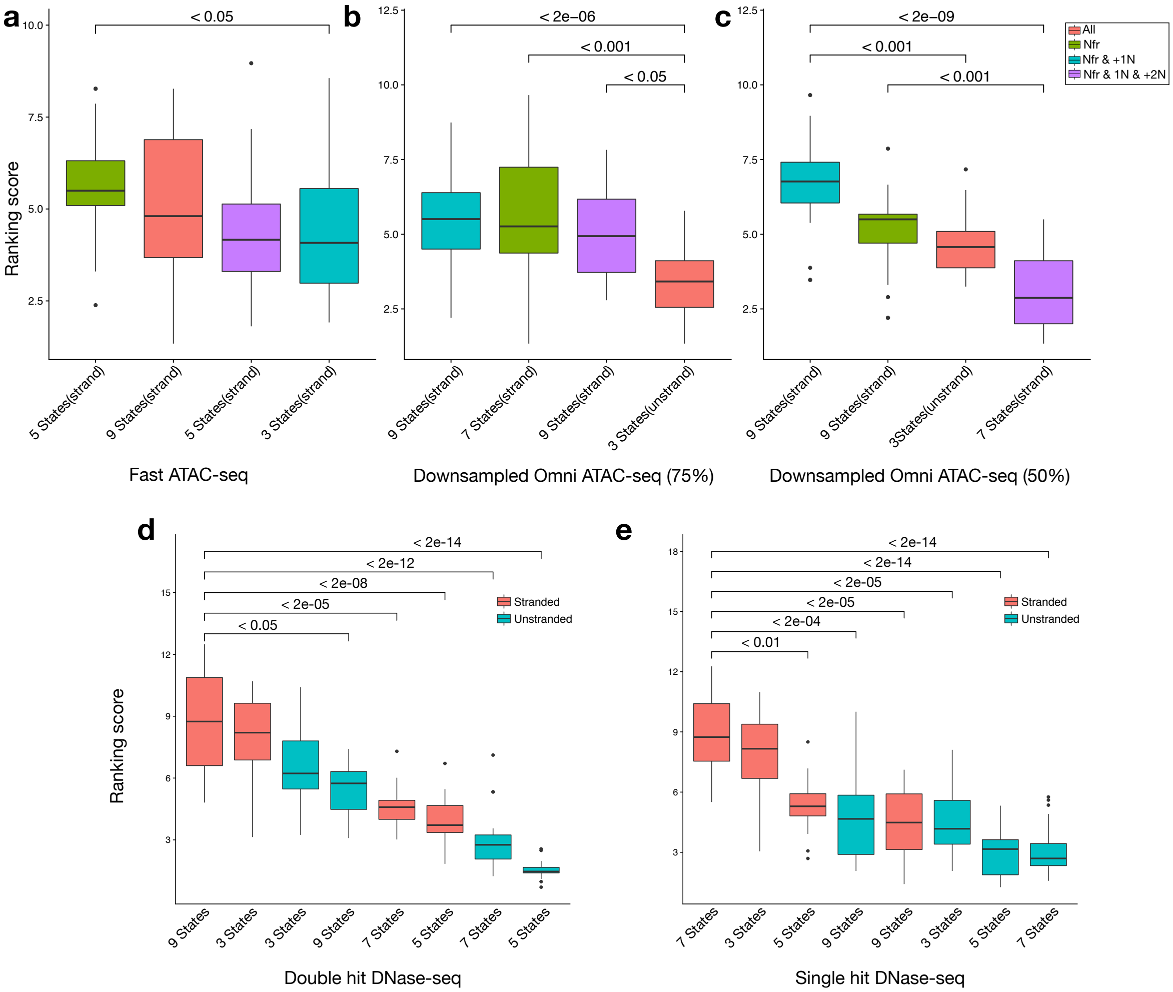

Figure S13: Ranking score contrasting the predictive performance of distinct signal decomposition strategies for Fast ATAC-seq (**a**) and downsampled Omni ATAC-seq (**b-c**) on GM12878 cells. Ranking score contrasting number of HMM states and the use of strand specific signals for data based on double hit DNase-seq (**d**) and single hit DNase-seq protocol (**e**). Nucleosome decomposition is not possible for DNase-seq given the lack of paired end DNase-seq libraries. The best models for each of this comparisons are used in further experiments for the given DNase/ATAC-seq protocol(see Table S17-S20 for complete results of statistics test).

a

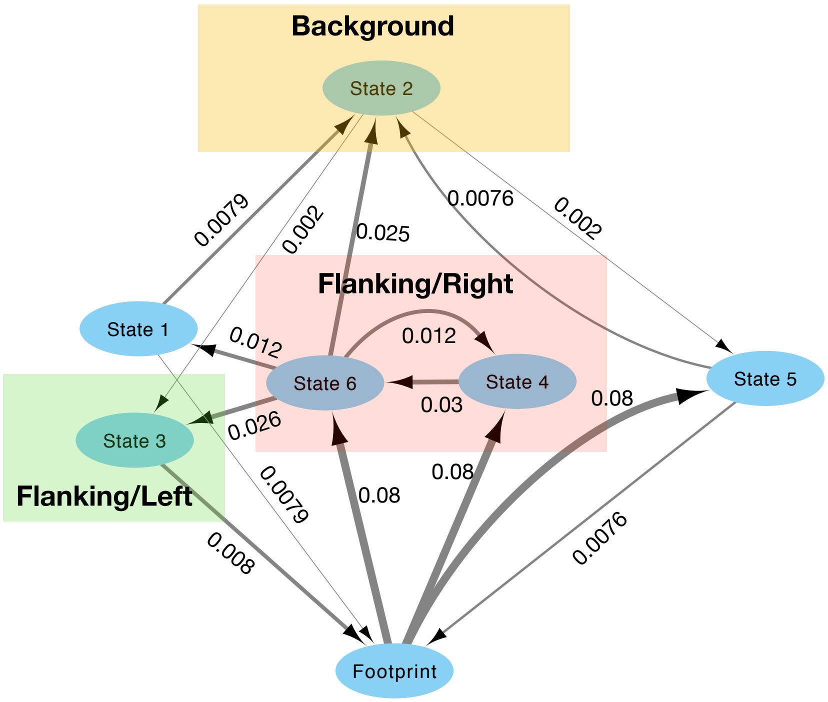

b

State (mean estimates)	Nfr Forward	Nfr Reversed	+1N Forward	+1N Reverse
State 2 (Background)	0.018	0.017	0.035	0.035
State 3 (Flanking Left)	**0.264**	0.040	0.058	**0.261**
State 4 (Flanking Right)	0.059	**0.458**	**0.451**	0.085
State 6 (Flanking Right)	0.034	**0.105**	**0.105**	0.043
Footprints	0.132	0.113	0.112	0.107
State 1	0.020	0.019	0.177	0.034
State 5	0.019	0.020	0.033	0.174

Figure S14: Transitions (**a**) probabilities and mean estimates (**b**) for the best HMM topology learned for Omni ATAC-seq. This HMM is based on 7 states and used strand specific `Nfr` and `+1N` decompositions as inputs. Self transitions are omitted. This HMM, which is trained using a semi-supervised approach with annotation of the footprint state, learns a state (2) for modelling background distribution with high self transition and low means for all signals, one state (3) associated to left flanking region with high `Nfr Forward` and `+1N Reverse` values and two states (4 and 6) associated to right flanking regions with high `Nfr Reverse` and `+1N Forward` values.

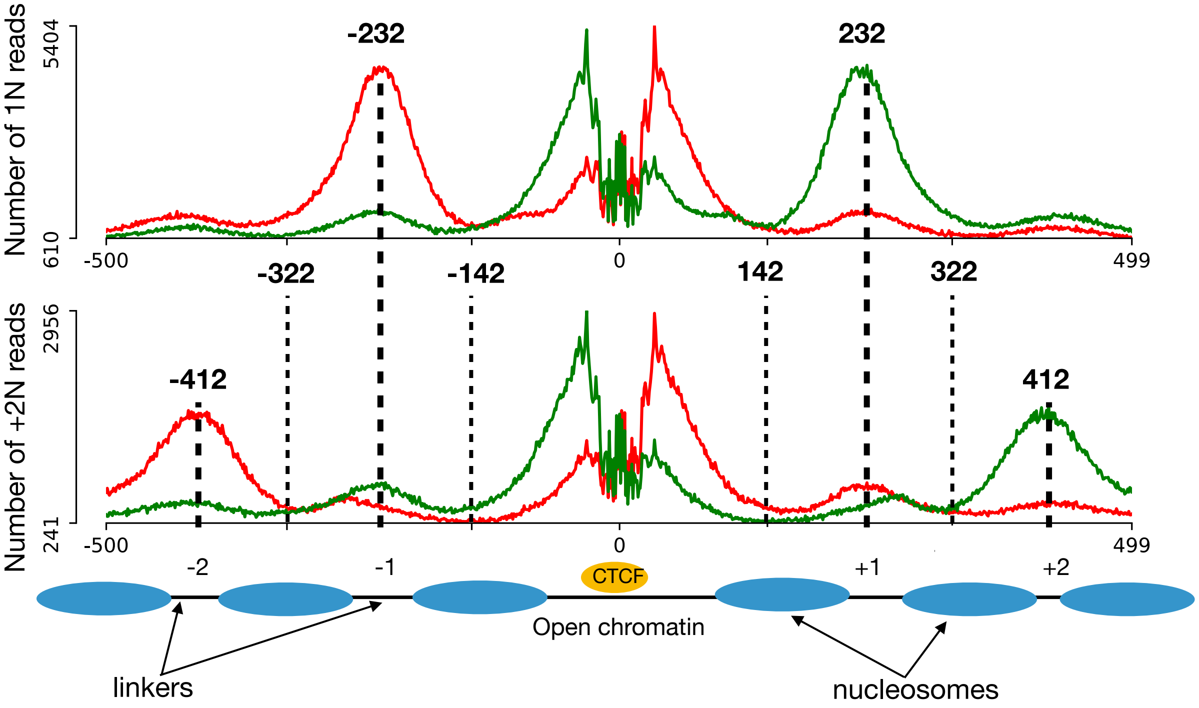

Figure S15: Calculation of the relative distance between binding site and the nucleosomes. We first estimate the position of -1, -2, +1, +2 linkers by searching for the local maximum values of smoothed 1N and +2N ATAC-seq signals. Given that the nucleosome core particle consists of approximately 146 base pairs (bp) of DNA, we then can estimate the linker size and finally obtain the relative distances.

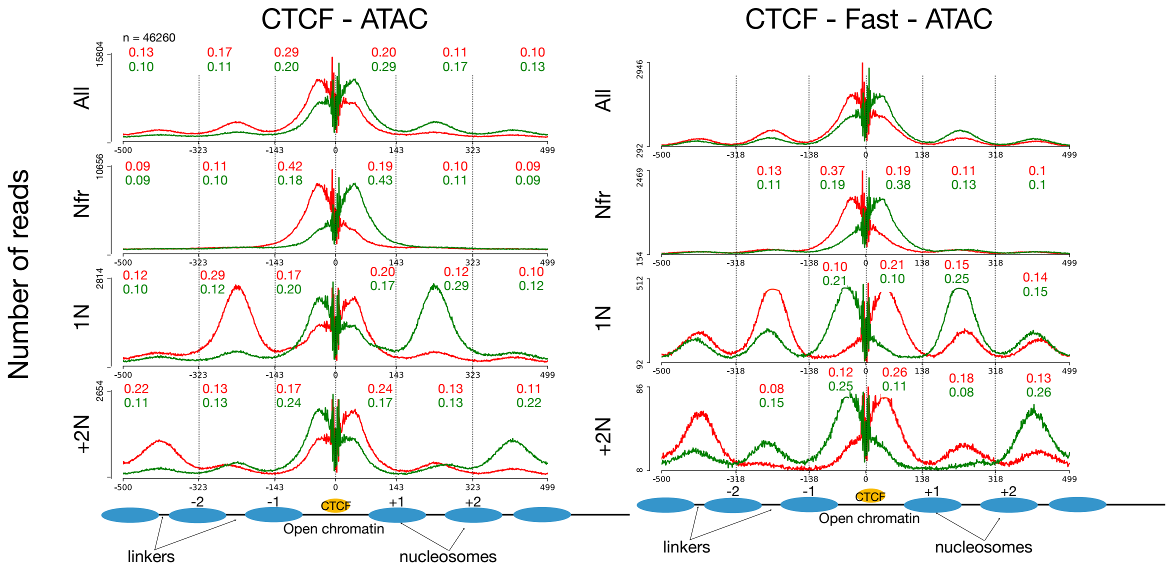

Figure S16: Strand specific and bias corrected cleavage signals for distinct fragment sizes around CTCF ChIP-seq peaks on GM12878 cells for standard and Fast ATAC-seq protocols. Strand patterns are similar to the ones in Omni-ATAC-seq (Main Fig. 4b).

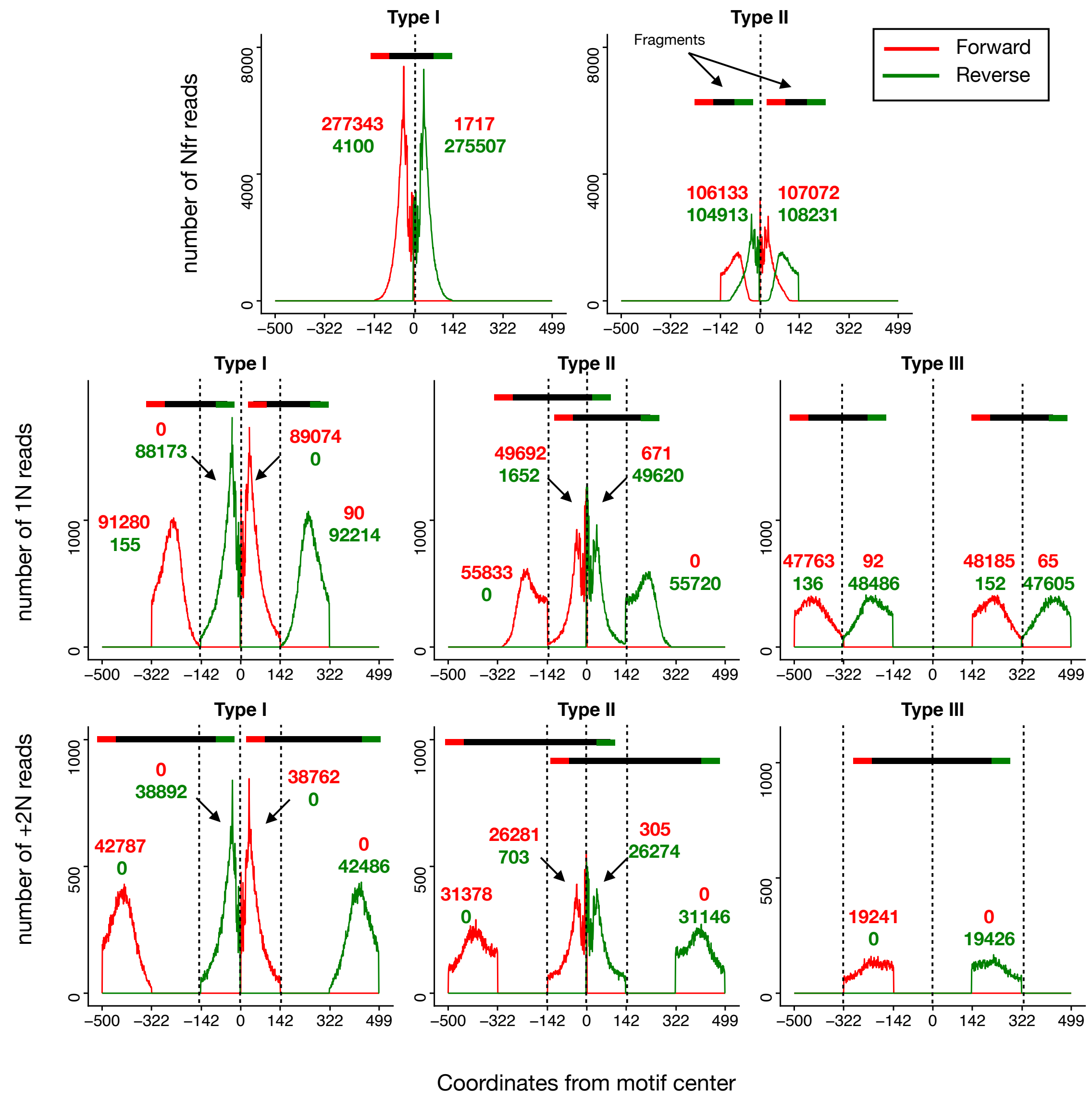

Figure S17: Number of reads (without bias correction) of standard ATAC-seq for each type of reads defined in Fig. 3A around CTCF ChIP-seq peaks on GM12878 cells. As for Omni-ATAC-seq, we observe more type I than type II reads for `Nfr` (281k vs 210k), `1N` (178k vs 105k) and +2N reads (81k vs. 57k). These supports the strand bias observed in nucleosome size decomposed ATAC-seq profiles.

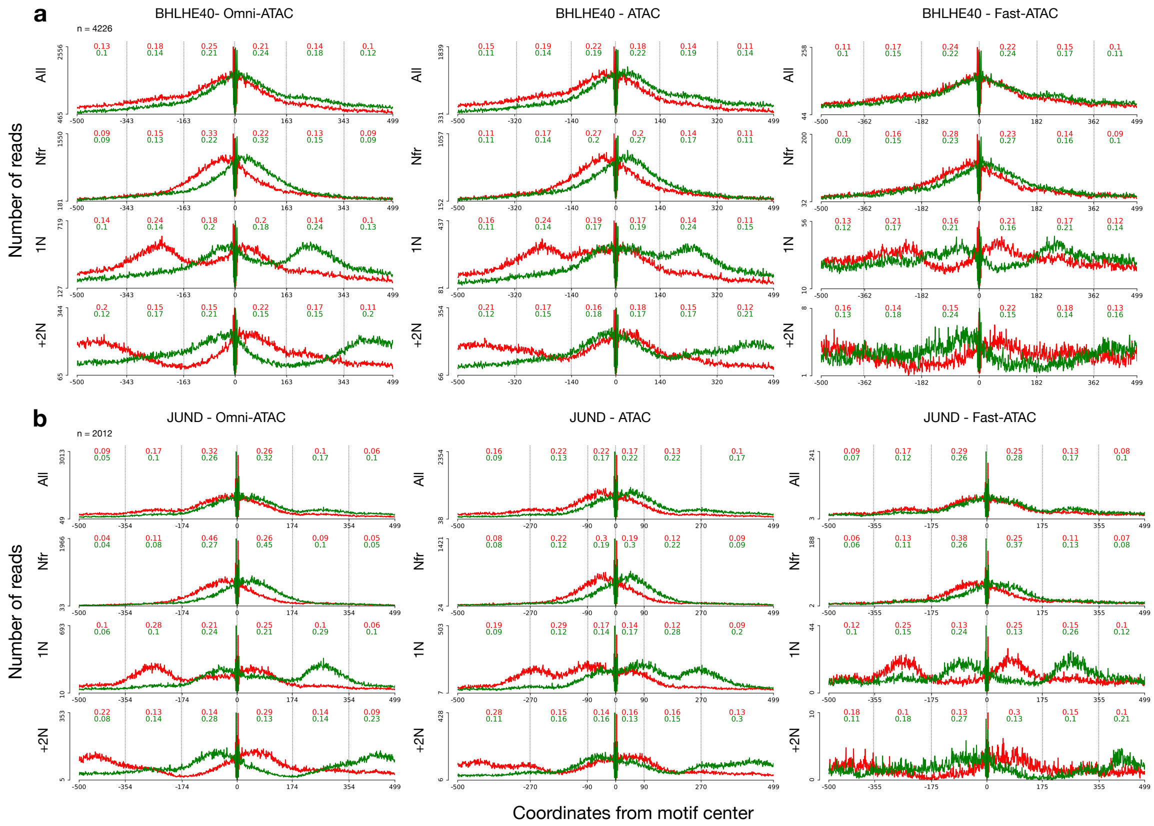

Figure S18: Average bias corrected cleavage signals of GM12878 cells from Omni, standard and Fast ATAC-seq protocols for factors BHLHE40 and JUND using different nucleosome decompositions.

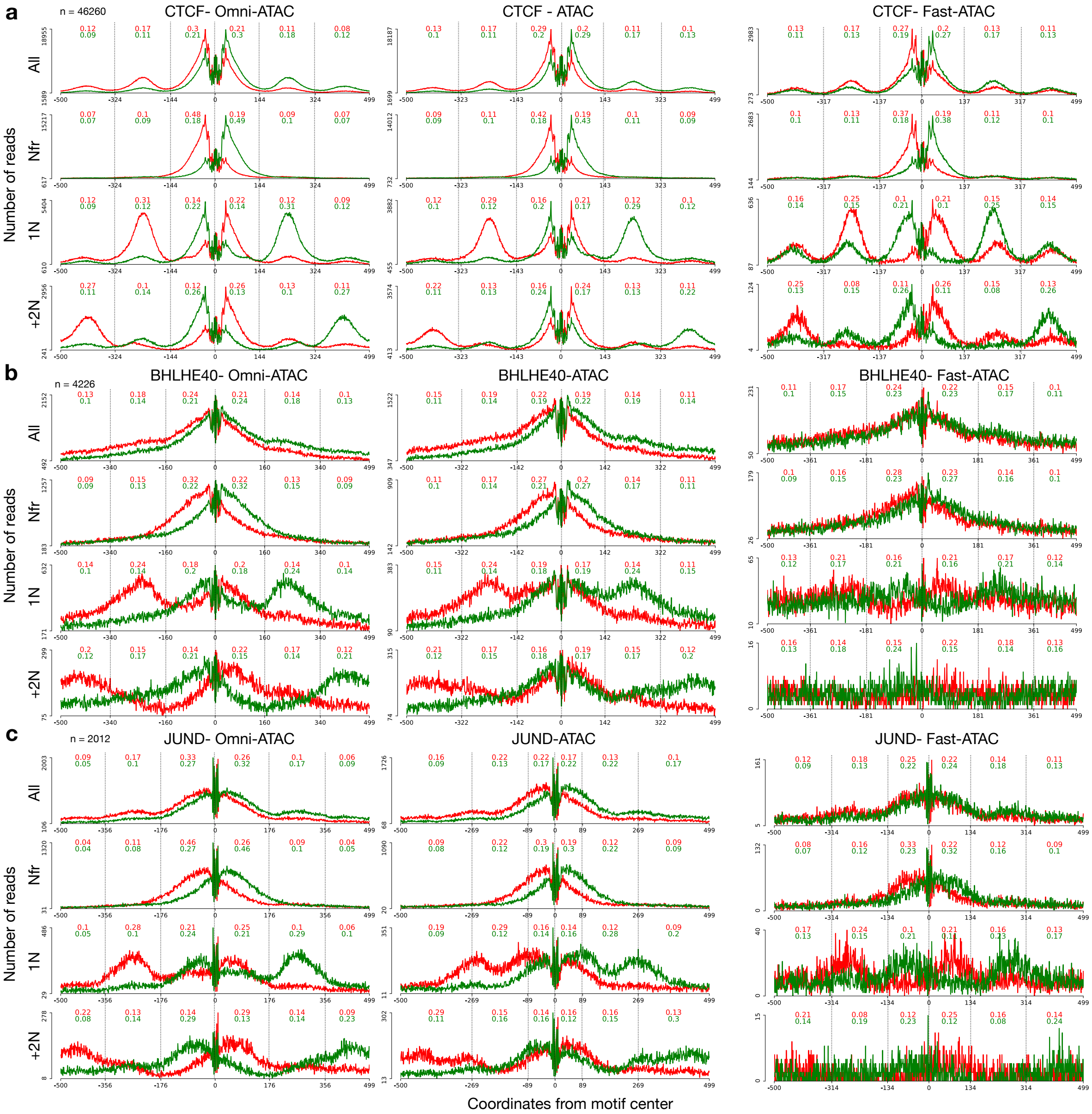

Figure S19: Average cleavage signals (without bias correction) of GM12878 cells from Omni, standard and Fast ATAC-seq protocols for factors CTCF, BHLHE40 and JUND using using different nucleosome decompositions.

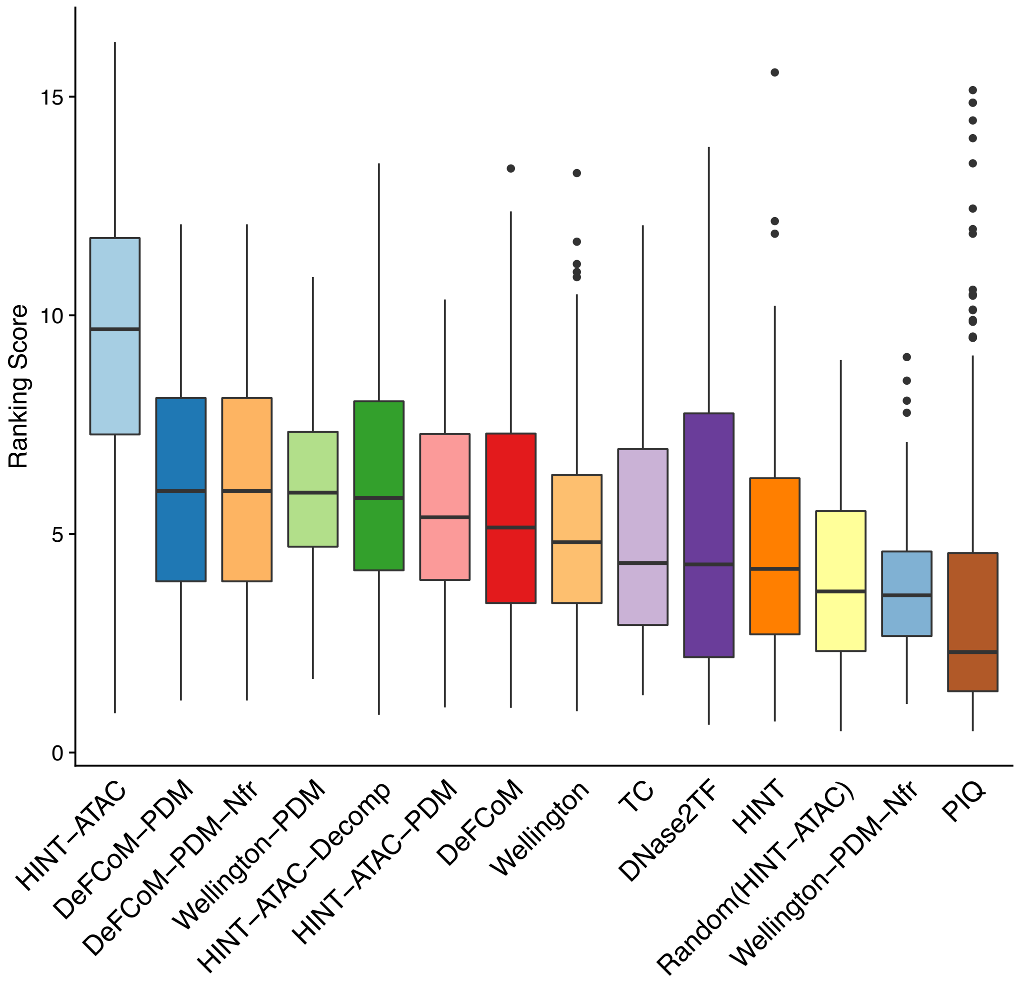

Figure S20: Extension of the comparative analysis (Fig. 5a - main manuscript) of distinct footprinting methods for Omni ATAC-seq on K562 cells and standard ATAC-seq on K562 and H1-ESC cells (see Table S21-S22 for statistics results). This comparison includes HINT-ATAC with both PDM bias correction and nucleosome decompositions (HINT-ATAC), HINT-ATAC with PDM bias correction and all reads (HINT-ATAC-PDM) and HINT-ATAC with nucleosome decomposition and no bias correction (HINT-ATAC-Decomp). While HINT-ATAC-PDM and HINT-ATAC-Decomp improve their performance in comparison to HINT, their combination (HINT-ATAC) results in the best ranked method. Competing methods Wellington and DeFCoM are also tested with both bias correction and the use of nucleosome free reads (Wellington-PDM-Nfr and DeFCoM-PDM-Nfr). While both methods profit from PDM bias correction, there is no improvement on the use of `Nfr` reads. We also include a control based on the execution of HINT-ATAC on K562 and H1-ESC libraries, where read locations were randomly shifted between 0 and 15 bps from its alignment position [Random(HINT-ATAC)]. As expected, most methods (with the exception of PIQ) have higher rankings than this baseline approach.

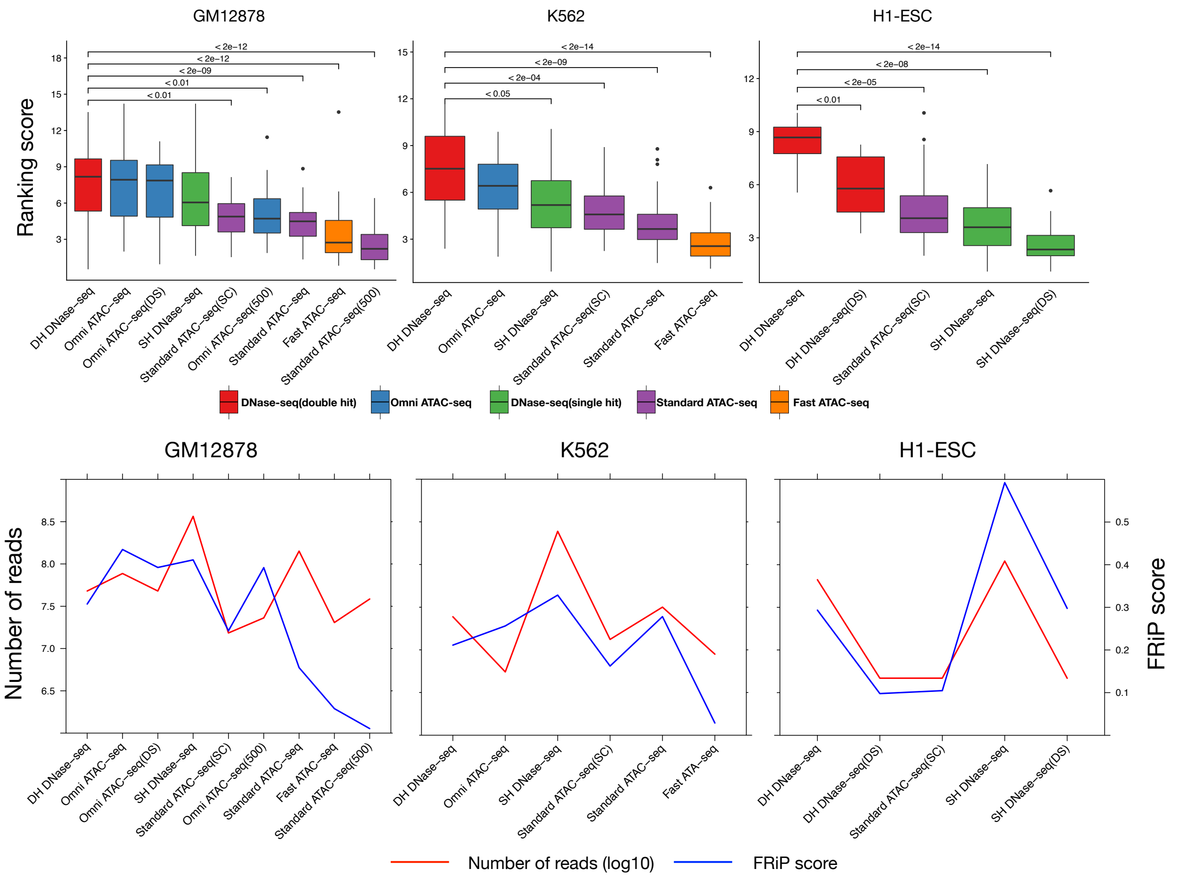

Figure S21: Comparison between different ATAC-seq and DNase-seq protocols in terms of footprint prediction for GM12878 (left), K562 (middle) and H1-ESC (right). DNase-seq data of GM12878, K562 and H1-ESC are based on either double hit ("DH") or single hit ("SH") protocols. ATAC-seq data was based on Omni, Fast and standard protocols. For each protocol, we used the best HMM and nucleosome decomposition strategy as trained/evaluated in GM12878 cells. Some of the standard ATAC-seq libraries were based on distinct number of cells (50.000 or 500) or performed on a single cell (SC) level. We have down-sampled Omni-ATAC [Omni-ATAC(DS)] to have a similar number of reads than DNase-SH for GM12878 and we have down-sampled DNase-DH [DNase-DH(DS)] to have the same number of reads as ATAC-seq in H1-ESC. While downsampling reduced scores, it did not affect the overall ranking of protocols. We observed that Omni ATAC-seq significantly outperformed the other protocols for GM12878 ($p-$value < 0.01) and K562 ($p-$value < 0.05), with exception of downsampled Omni and single hit DNase-seq. For H1-ESC, downsampled DNase-seq (double hit) had the second highest ranking score and significantly outperformed the original and downsampled single hit DNase-seq ($p-$value < 0.05). See Table S25-S30 for statistics test results).

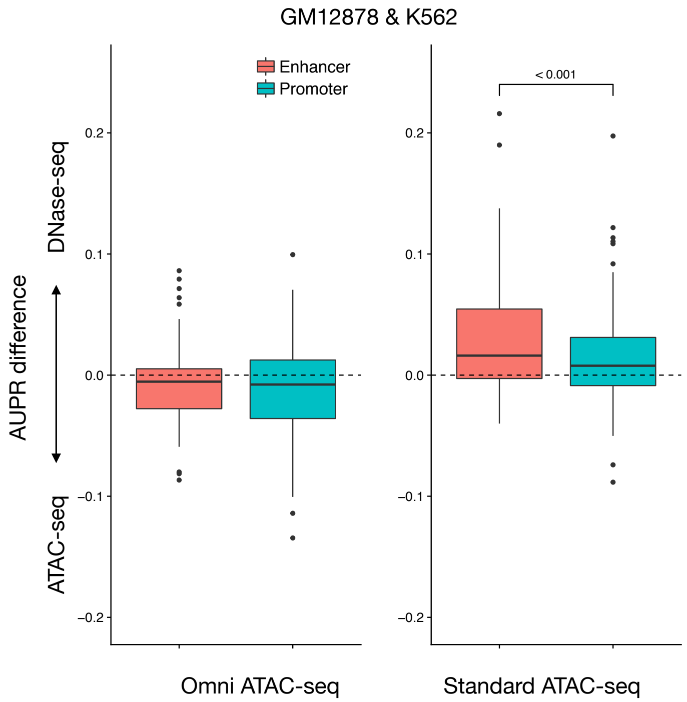

Figure S22: Difference in AUPR of double hit DNase-seq vs. Omni ATAC-seq (left) and double hit DNase-seq vs. standard ATAC-seq (right) on either enhancer and promoter regions. We observe positive values (higher AUPR for DNase-seq than standard ATAC-seq) in enhancer regions, but no significant differences are found on comparing Omni-ATAC and DNase-seq (Wilcoxon signed rank test).

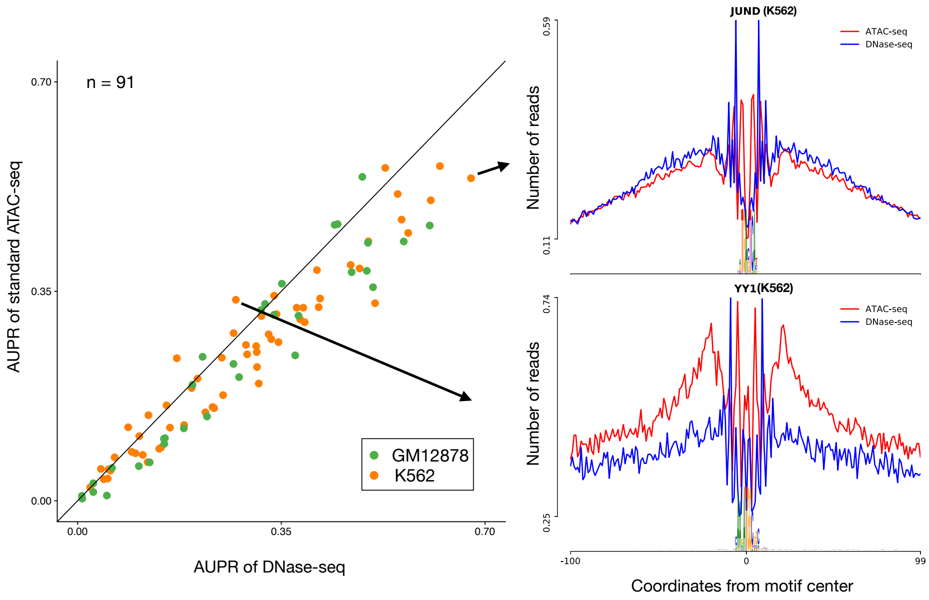

Figure S23: AUPR values of DNase-seq (double hit) and standard ATAC-seq for 91 factors in K562 and GM12878 cells. We highlight the footprint profiles of two factors (YY1 and JUND) with high differences in AUPR as in Fig. 5b of the main text.

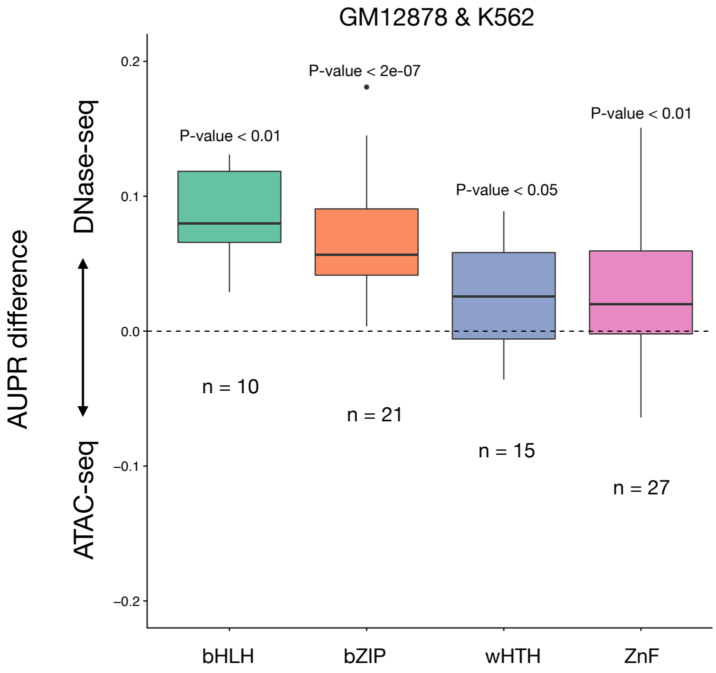

Figure S24: AUPR differences (DNase-ATAC) of double hit DNase-seq vs standard ATAC-seq based on different transcription factor families. p-value are obtained by a t-test (mean different from zero).

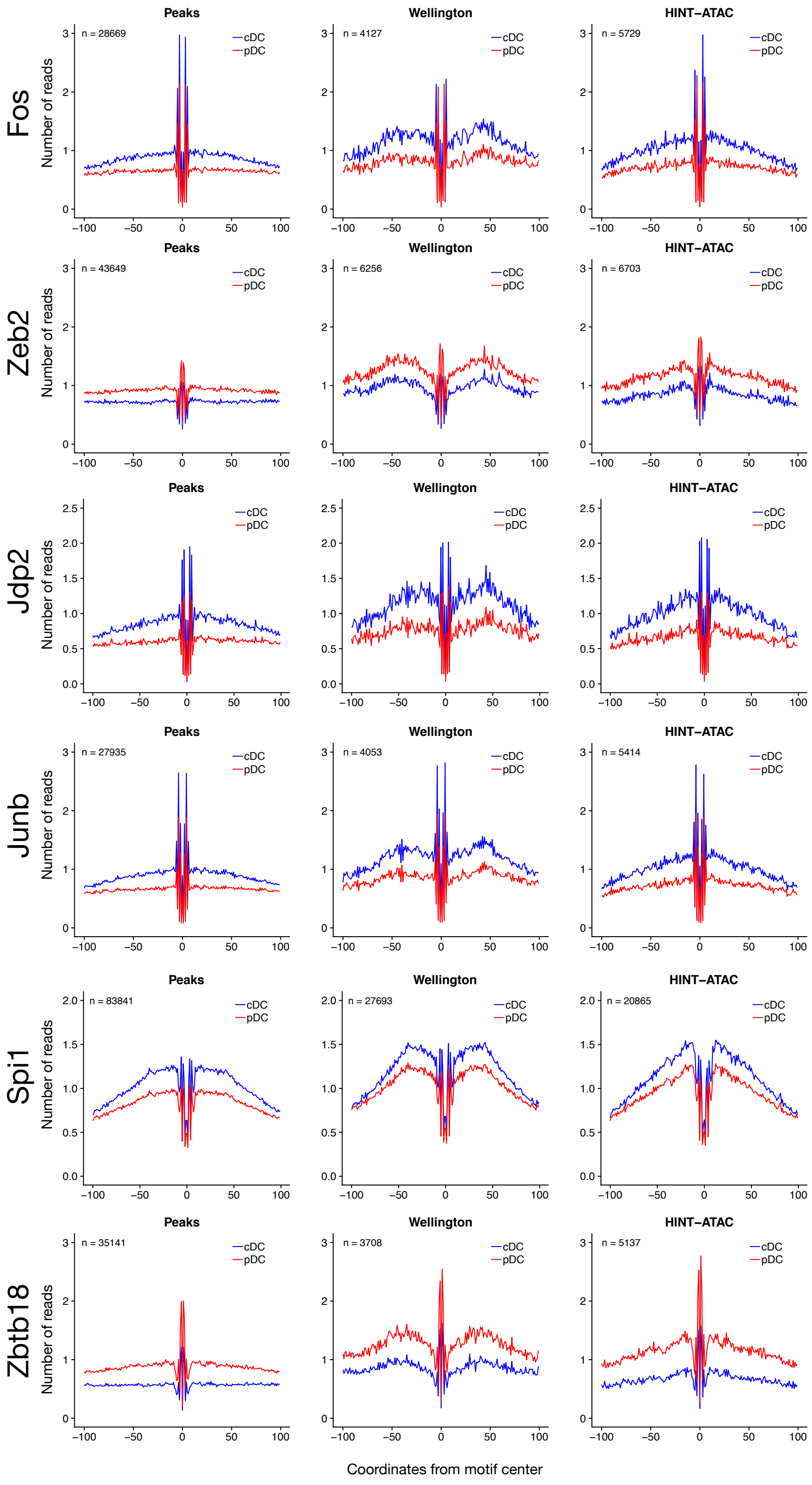

Figure S25: Average bias corrected cleavage profiles of motif predicted binding sites for Fos, Zeb2, Jdp2, Junb, Spi1 and Zbtb18 inside ATAC-seq peaks, or supported by footprints of Wellington and HINT-ATAC.

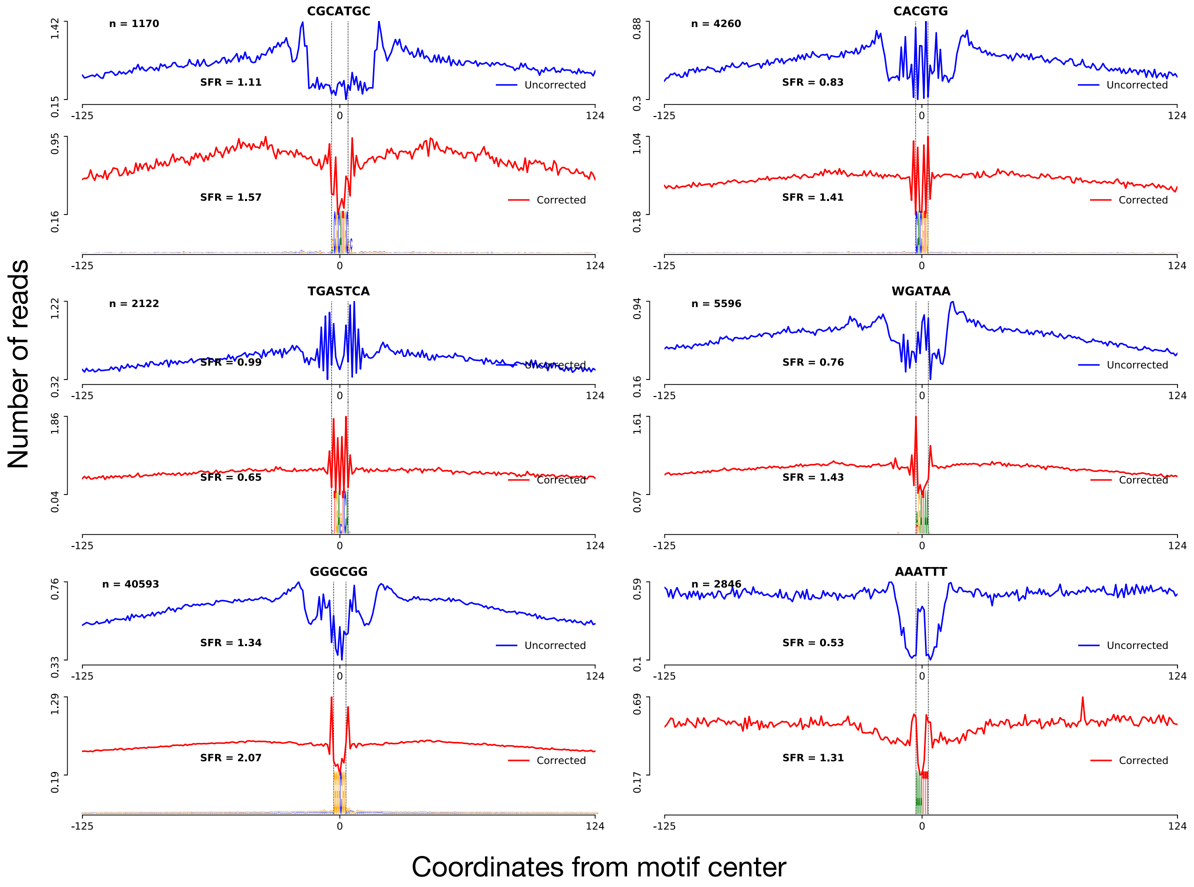

Figure S26: PDM-based bias corrected (red) and uncorrected (blue) on ATAC-seq of primary erythroid samples (Schwessinger et al. 2017). We show here the same k-mers as in supplemental figure S7 of (Schwessinger et al. 2017), which all have clear footprint profiles for DNase-seq, but not for ATAC-seq. We observe that for 5 out of the 6 motifs, PDM-based bias correction leaves clear footprints encompassing only the conserved DNA sequences of the motifs. To make this point more quantitative, we calculated the shoulder-footprint-ratio (SFR) proposed in Schwessinger et al. (2017), where values higher than 1 indicate the presence of a footprint. We observe higher SFR values for all motifs with the exception of TGASTCA.

Table S1: Average ranking score of bias estimation methods for standard ATAC-seq in GM12787 cells.

**Average ranking score**	
PDM(8)	7.135296
KMER(12)	6.759167
PDM(8)-Naked	6.088727
KMER(12)-Naked	5.444305
PWM(8)	4.399636
Uncorrected	3.199035
PWM(8)-Naked	3.052238

Table S2: Friedman-Nemenyi test results of the **Ranking Score** metric of bias estimation methods for standard ATAC-seq in GM12787 cells. The asterisk and the two asterisks, respectively, mean that the method in the column outperformed the method in the row with significance levels of 0.05 and 0.01

	PDM(8)	KMER(12)	PDM(8)-Naked	KMER(12)-Naked	PWM(8)	Uncorrected	PWM(8)-Naked
PDM(8)							
KMER(12)							
PDM(8)-Naked							
KMER(12)-Naked	*						
PWM(8)	**	**	*				
Uncorrected	**	**	**	**			
PWM(8)-Naked	**	**	**	**			

Table S3: Average ranking score of bias estimation methods for DNase-seq in GM12878 cells.

**Average ranking score**	
PDM(8)	6.461679
PDM(8)-Naked	5.943931
PWM(4)	5.762482
PWM(4)-Naked	5.673683
KMER(4)	5.529089
KMER(4)-Naked	4.602449
Uncorrected	2.084486

Table S4: Friedman-Nemenyi test results of the **Ranking Score** metric for bias estimation methods for DNase-seq in GM12878 cells. The asterisk and the two asterisks, respectively, mean that the method in the column outperformed the method in the row with significance levels of 0.05 and 0.01

	PDM(8)	PDM(8)-Naked	PWM(4)	PWM(4)-Naked	KMER(4)	KMER(4)-Naked	Uncorrected
PDM(8)							
PDM(8)-Naked							
PWM(4)							
PWM(4)-Naked							
KMER(4)							
KMER(4)-Naked							
Uncorrected	**	**	**	**	**	**	

Table S5: Average ranking score of bias estimation methods for Omni ATAC-seq in GM12878 cells.

**Average ranking score**	
KMER(12)	7.149596
PDM(8)	6.338731
PWM(4)	2.968498
Uncorrected	3.055695

Table S6: Friedman-Nemenyi test results of the **Ranking Score** metric for bias estimation methods for Omni ATAC-seq in GM12878 cells. The asterisk and the two asterisks, respectively, mean that the method in the column outperformed the method in the row with significance levels of 0.05 and 0.01

	KMER(12)	PDM(8)	PWM(4)	Uncorrected
KMER(12)				
PDM(8)				
PWM(4)	**	**		
Uncorrected	**	**		

Table S7: Average ranking score of bias estimation methods for Fast ATAC-seq in GM12878 cells.

**Average ranking score**	
PDM(8)	5.901833
KMER(10)	4.796283
PWM(2)	5.201683
Uncorrected	3.612721

Table S8: Friedman-Nemenyi test results of the **Ranking Score** metric for bias estimation methods for Fast ATAC-seq in GM12878 cells. The asterisk and the two asterisks, respectively, mean that the method in the column outperformed the method in the row with significance levels of 0.05 and 0.01

	PDM(8)	KMER(10)	PWM(2)	Uncorrected
PDM(8)				
KMER(10)				
PWM(2)				
Uncorrected	**			

Table S9: Average ranking score of bias estimation methods for downsampled Omni ATAC-seq (75%) in GM12878 cells.

**Average ranking score**	
PDM(8)	8.286579
KMER(10)	5.075464
PWM(4)	3.383311
Uncorrected	2.767166

Table S10: Friedman-Nemenyi test results of the **Ranking Score** metric for bias estimation methods for downsampled Omni ATAC-seq (75%) in GM12878 cells. The asterisk and the two asterisks, respectively, mean that the method in the column outperformed the method in the row with significance levels of 0.05 and 0.01

	PDM(8)	KMER(10)	PWM(4)	Uncorrected
PDM(8)				
KMER(10)	*			
PWM(4)	**			
Uncorrected	**	**		

Table S11: Average ranking score of bias estimation methods for downsampled Omni ATAC-seq (50%) in GM12878 cells.

**Average ranking score**	
PDM(8)	8.283322
KMER(10)	4.947127
PWM(2)	3.451975
Uncorrected	2.826416

Table S12: Friedman-Nemenyi test results of the **Ranking Score** metric for for downsampled Omni ATAC-seq (50%) in GM12878 cells. The asterisk and the two asterisks, respectively, mean that the method in the column outperformed the method in the row with significance levels of 0.05 and 0.01

	PDM(8)	KMER(10)	PWM(2)	Uncorrected
PDM(8)				
KMER(10)	**			
PWM(2)	**	*		
Uncorrected	**	**		

Table S13: Average ranking score of the best models of Omni ATAC-seq in GM12878 cells

**Average ranking score**	
Nfr & +1N	7.156957
Nfr	7.081356
All	3.651808
Nfr & 1N & +2N	1.622399

Table S14: Friedman-Nemenyi test results of the **Ranking Score** metric for the best models of Omni ATAC-seq in GM12878 cells. The asterisk and the two asterisks, respectively, mean that the method in the column outperformed the method in the row with significance levels of 0.05 and 0.01

	Nfr & +1N	Nfr	All	Nfr & 1N & +2N
Nfr & +1N				
Nfr				
All	**	**		
Nfr & 1N & +2N	**	**	**	

Table S15: Average ranking score of the best models of standard ATAC-seq in GM12878 cells.

**Average ranking score**	
Nfr	6.930935
Nfr & +1N	4.579251
All	4.113331
Nfr & 1N & +2N	3.889003

Table S16: Friedman-Nemenyi test results of the **Ranking Score** metric the best models of standard ATAC-seq in GM12878 cells. The asterisk and the two asterisks, respectively, mean that the method in the column outperformed the method in the row with significance levels of 0.05 and 0.01

	Nfr	Nfr & +1N	All	Nfr & 1N & +2N
Nfr				
Nfr & +1N	**			
All	**			
Nfr & 1N & +2N	**			

Table S17: Average ranking score of methods for double hit DNase-seq in GM12878 cells

**Average Ranking Score**	
Stranded-9 states	8.672999
Stranded-3 states	8.073677
Unstranded-3 states	6.634533
Unstranded-9 states	5.442201
Stranded-7 states	4.583651
Stranded-5 states	3.924278
Unstranded-7 states	2.845278
Unstranded-5 states	1.528667

Table S18: Friedman-Nemenyi test results of the **Ranking Score** metric for double hit DNase-seq in GM12878 cells. The asterisk and the two asterisks, respectively, mean that the method in the column outperformed the method in the row with significance levels of 0.05 and 0.01

	Stranded-9 states	Stranded-3 states	Unstranded-3 states	Unstranded-9 states	Stranded-7 states	Stranded-5 states	Unstranded-7 states	Unstranded-5 states
Stranded-9 states								
Stranded-3 states								
Unstranded-3 states								
Unstranded-9 states	*	*						
Stranded-7 states	**	**						
Stranded-5 states	**	**	**					
Unstranded-7 states	**	**	**	**				
Unstranded-5 states	**	**	**	**	**	**		

Table S19: Average ranking score of methods for single hit DNase-seq in GM12878 cells

**Average Ranking Score**	
Stranded-7 states	8.872965
Stranded-3 states	7.750739
Stranded-5 states	5.369318
Unstranded-9 states	4.662625
Stranded-9 states	4.553709
Unstranded-3 states	4.576396
Unstranded-5 states	2.825925
Unstranded-7 states	3.090412

Table S20: Friedman-Nemenyi test results of the **Ranking Score** metric for single hit DNase-seq in GM12878 cells. The asterisk and the two asterisks, respectively, mean that the method in the column outperformed the method in the row with significance levels of 0.05 and 0.01

	Stranded-7 states	Stranded-3 states	Stranded-5 states	Unstranded-9 states	Stranded-9 states	Unstranded-3 states	Unstranded-5 states	Unstranded-7 states
Stranded-7 states								
Stranded-3 states								
Stranded-5 states	**							
Unstranded-9 states	**	**						
Stranded-9 states	**	**						
Unstranded-3 states	**	**						
Unstranded-5 states	**	**	**	*				
Unstranded-7 states	**	**	**	*				

Table S21: Average ranking score of comparative evaluation of competing methods using the `Test dataset` (K562 and H1-ESC cells).

**Average ranking score**	
HINT-ATAC	9.399456
DeFCoM-PDM	5.968795
DeFCoM-PDM-Nfr	5.968795
Wellington-PDM	6.066458
HINT-ATAC-Decomp	6.075212
HINT-ATAC-PDM	5.517628
DeFCoM	5.525881
Wellington	5.176601
TC	5.180950
DNase2TF	5.070137
HINT	4.667121
Random(HINT-ATAC)	3.860066
Wellington-PDM-Nfr	3.829496
PIQ	3.866870

Table S22: Friedman-Nemenyi test results of the **Ranking Score** metric for comparative evaluation of competing methods using the `Test dataset` (K562 and H1-ESC cells). The asterisk and the two asterisks, respectively, mean that the method in the column outperformed the method in the row with significance levels of 0.05 and 0.01

	HINT-ATAC	DeFCoM-PDM	DeFCoM-PDM-Nfr	Wellington-PDM	HINT-ATAC-Decomp	HINT-ATAC-PDM	DeFCoM	Wellington	TC	DNase2TF	HINTBC	Random(HINT-ATAC)	Wellington-PDM-Nfr	PIQ
HINT-ATAC														
DeFCoM-PDM	**													
DeFCoM-PDM-Nfr	**													
Wellington-PDM	**													
HINT-ATAC-Decomp	**													
HINT-ATAC-PDM	**													
DeFCoM	**													
Wellington	**													
TC	**													
DNase2TF	**			*										
HINTBC	**	*	*	*	*									
Random(HINT-ATAC)	**	**	**	**	**	**	**	*	*					
Wellington-PDM-Nfr	**	**	**	**	**	**	**	**	**	*				
PIQ	**	**	**	**	**	**	**	**	**	**	*			

Table S23: Average ranking score of comparative evaluation of competing methods using the `Test dataset` (K562 and H1-ESC cells).

**Average ranking score**	
HINT-ATAC	8.378000
Wellington-PDM	5.866785
DeFCoM-PDM	5.731028
DeFCoM	5.107398
Wellington	4.970716
TC	4.765912
DNase2TF	4.654832
HINTBC	4.447350
PIQ	3.583616

Table S24: Friedman-Nemenyi test results of the **Ranking Score** metric for comparative evaluation of competing methods using the `Test dataset` (K562 and H1-ESC cells). The asterisk and the two asterisks, respectively, mean that the method in the column outperformed the method in the row with significance levels of 0.05 and 0.01

	HINT-ATAC	Wellington-PDM	DeFCoM-PDM	DeFCoM	Wellington	TC	DNase2TF	HINTBC	PIQ
HINT-ATAC									
DeFCoM-PDM	**								
Wellington-PDM	**								
DeFCoM	**								
Wellington	**								
TC	**	*							
DNase2TF	**	*	*						
HINTBC	**	**	**						
PIQ	**	**	**	**	**	**	**	**	

Table S25: Average ranking score of different protocols for GM12878 cells.

**Average Ranking Score**	
DU DNase-seq	7.808499
Omni ATAC-seq	7.654787
Omni ATAC-seq(DS)	7.115633
SH DNase-seq	6.682315
Standard ATAC-seq	4.508517
Omni ATAC-seq(500)	4.461801
Standard ATAC-seq	4.088874
Fast ATAC-seq	2.936969
Standard ATAC-seq(500)	2.118225

Table S26: Friedman-Nemenyi test results of the **Ranking Score** metric for GM12878 cells.. The asterisk and the two asterisks, respectively, mean that the method in the column outperformed the method in the row with significance levels of 0.05 and 0.01

	DH DNase-seq	Omni ATAC-seq	Omni ATAC-seq(DS)	SH DNase-seq	Standard ATAC-seq(SC)	Omni ATAC-seq(500)	Standard ATAC-seq	Fast ATAC-seq	Standard ATAC-seq(500)
DH DNase-seq									
Omni ATAC-seq									
Omni ATAC-seq(DS)									
SH DNase-seq									
Standard ATAC-seq(SC)	**	**	*						
Omni ATAC-seq(500)	**	**	*						
Standard ATAC-seq	**	**	**	*					
Fast ATAC-seq	**	**	**	**					
Standard ATAC-seq(500)	**	**	**	**	**	**	*		

Table S27: Average ranking score of different protocols for K562 cells.

**Average Ranking Score**	
DH DNase-seq	7.451539
Omni ATAC-seq	6.32159
SH DNase-seq	5.290864
Standard ATAC-seq(SC)	4.775758
Standard ATAC-seq	3.914304
Fast ATAC-seq	2.823203

Table S28: Friedman-Nemenyi test results of the **Ranking Score** metric for K562 cells. The asterisk and the two asterisks, respectively, mean that the method in the column outperformed the method in the row with significance levels of 0.05 and 0.01

	DH DNase-seq	Omni ATAC-seq	SH DNase-seq	Standard ATAC-seq(SC)	Standard ATAC-seq	Fast ATAC-seq
DH DNase-seq						
Omni ATAC-seq						
SH DNase-seq	*					
Standard ATAC-seq(SC)	**	*				
Standard ATAC-seq	**	**	*			
Fast ATAC-seq	**	**	**	**	*	

Table S29: Average ranking score of different protocols for H1-ESC cells.

**Average Ranking Score**	
DH DNase-seq	8.400458
DH DNase-seq(DS)	5.803238
Standard ATAC-seq(SC)	4.563197
SH DNase-seq	3.657482
SH DNase-seq(DS)	2.557938

Table S30: Friedman-Nemenyi test results of the **Ranking Score** metric for H1-ESC cells. The asterisk and the two asterisks, respectively, mean that the method in the column outperformed the method in the row with significance levels of 0.05 and 0.01

	DH DNase-seq	DH DNase-seq(DS)	Standard ATAC-seq(SC)	SH DNase-seq	SH DNase-seq(DS)
DH DNase-seq					
DH DNase-seq(DS)	**				
Standard ATAC-seq(SC)	**				
SH DNase-seq	**	*			
SH DNase-seq(DS)	**	**	**		

Table S31: **Position frequency matrices (PFMs) and transcription factors (TFs) ChIP-seq used in the ChIP-seq evaluation methodology.** ChIP-seq was obtained from multiple labs within the Encode Consortium (2012). PFMs were obtained from Jaspar (Mathelier et al. 2014), Uniprobe (Robasky and Bulyk 2011) and Transfac (Matys et al. 2006).

Cell	Factor	PFM Repository	PFM ID	ChIP-seq ID	# Motifs	# Peaks	# Motifs with Peaks
K562	ARID3A	Jaspar	MA0151.1	wgEncodeEH002861	2112327	9026	650
K562	ATF1	Uniprobe	UP00020	wgEncodeEH002865	246442	14864	5167
K562	ATF3	Jaspar	MA0018.2	wgEncodeEH000700	496476	1233	277
K562	BACH1	Transfac	M00495	wgEncodeEH002846	614421	3806	2322
K562	BHLHE40	Jaspar	MA0464.1	wgEncodeEH001857	572185	22497	7994
K562	CCNT2	Jaspar	MA0140.2	wgEncodeEH001864	708983	20057	2537
K562	CEBPB	Jaspar	MA0466.1	wgEncodeEH001821	1342548	38715	26843
K562	CTCF	Jaspar	MA0139.1	wgEncodeEH002797	565933	54387	45170
K562	CTCFL	Jaspar	MA0139.1	wgEncodeEH001652	565933	11533	10157
K562	E2F4	Jaspar	MA0470.1	wgEncodeEH000671	173646	8181	3351
K562	E2F6	Jaspar	MA0471.1	wgEncodeEH000676	1051116	16312	5022
K562	EFOS	Jaspar	MA0476.1	wgEncodeEH001207	762222	10256	16616
K562	EGATA	Jaspar	MA0036.2	wgEncodeEH001208	1028569	11478	4341
K562	EGR1	Jaspar	MA0162.2	wgEncodeEH001646	1060314	36997	39583
K562	EJUNB	Jaspar	MA0490.1	wgEncodeEH001210	717235	12287	14611
K562	EJUND	Jaspar	MA0491.1	wgEncodeEH001211	717223	26674	21453
K562	ELF1	Jaspar	MA0473.1	wgEncodeEH001619	1026618	27780	16016
K562	ELK1	Jaspar	MA0028.1	wgEncodeEH003356	100691	2961	1592
K562	ETS1	Jaspar	MA0098.2	wgEncodeEH001580	1319961	10726	1966
K562	FOS	Jaspar	MA0476.1	wgEncodeEH000619	762222	7646	6847
K562	FOSL1	Jaspar	MA0477.1	wgEncodeEH001637	699220	11174	16921
K562	GABP	Jaspar	MA0062.2	wgEncodeEH001604	181503	14393	6687
K562	GATA1	Jaspar	MA0035.3	wgEncodeEH000638	1040470	4074	2164
K562	GATA2	Jaspar	MA0036.2	wgEncodeEH000683	1028569	10648	4681
K562	IRF1	Jaspar	MA0050.2	wgEncodeEH002798	2330047	8352	5027
K562	JUN	Jaspar	MA0488.1	wgEncodeEH000620	832374	9848	2939
K562	JUND	Jaspar	MA0491.1	wgEncodeEH002164	717223	40052	29599
K562	MAFF	Jaspar	MA0495.1	wgEncodeEH002804	1215808	25074	20563
K562	MAFK	Jaspar	MA0496.1	wgEncodeEH001844	1221488	19317	14001
K562	MAX	Jaspar	MA0058.2	wgEncodeEH002869	855374	31436	5239
K562	MEF2A	Jaspar	MA0052.2	wgEncodeEH001663	3210613	5631	3068
K562	MYC	Jaspar	MA0147.2	wgEncodeEH000621	614797	5023	1698
K562	NFE2	Jaspar	MA0501.1	wgEncodeEH000624	796063	2637	2499
K562	NFYA	Jaspar	MA0060.2	wgEncodeEH002021	428913	4286	4220
K562	NFYB	Jaspar	MA0502.1	wgEncodeEH002024	470725	10096	12693
K562	NR2F2	Uniprobe	UP00009	wgEncodeEH002382	626663	16678	3289
K562	NRF1	Jaspar	MA0506.1	wgEncodeEH001796	137117	4211	6678
K562	PU1	Jaspar	MA0080.3	wgEncodeEH001482	2040890	28677	29997
K562	RAD21	Jaspar	MA0139.1	wgEncodeEH000649	565933	17627	17730
K562	REST	Jaspar	MA0138.2	wgEncodeEH001638	629168	15849	4543
K562	RFX5	Jaspar	MA0510.1	wgEncodeEH002033	629248	2201	519
K562	SIX5	Jaspar	MA0088.1	wgEncodeEH001483	1032447	4194	1792
K562	SMC3	Jaspar	MA0139.1	wgEncodeEH001845	565933	23598	22980

Cell	Factor	PFM Repository	PFM ID	ChIP-seq ID	# Motifs	# Peaks	# Motifs with Peaks
K562	SP1	Jaspar	MA0079.3	wgEncodeEH001578	1797400	7206	4932
K562	SP2	Jaspar	MA0516.1	wgEncodeEH001653	1587339	3124	2572
K562	SRF	Jaspar	MA0083.2	wgEncodeEH001600	1024023	4717	2617
K562	STAT1	Jaspar	MA0137.3	wgEncodeEH000664	1272026	1476	295
K562	STAT2	Jaspar	MA0517.1	wgEncodeEH000666	3077582	1923	1629
K562	STAT5A	Jaspar	MA0519.1	wgEncodeEH002347	1292097	9811	3776
K562	TAL1	Jaspar	MA0140.2	wgEncodeEH001824	708983	26260	12380
K562	TBP	Jaspar	MA0108.1	wgEncodeEH001825	834532	17558	480
K562	THAP1	Jaspar	MA0597.1	wgEncodeEH001655	561707	3506	349
K562	TR4	Jaspar	MA0504.1	wgEncodeEH000682	825980	587	262
K562	USF1	Jaspar	MA0093.2	wgEncodeEH001583	691899	18521	17111
K562	USF2	Jaspar	MA0526.1	wgEncodeEH001797	759040	3083	3515
K562	YY1	Jaspar	MA0095.2	wgEncodeEH000684	1325447	4948	3422
K562	ZBTB33	Jaspar	MA0527.1	wgEncodeEH001569	82928	3285	2847
K562	ZBTB7A	Uniprobe	UP00047	wgEncodeEH001620	412506	21711	948
K562	ZNF143	Jaspar	MA0088.1	wgEncodeEH002030	1032447	29069	4019
K562	ZNF263	Jaspar	MA0528.1	wgEncodeEH000630	2577084	3081	6388
GM12878	ATF3	Jaspar	MA0018.2	wgEncodeEH001562	496476	1677	272
GM12878	BHLHE40	Jaspar	MA0464.1	wgEncodeEH002025	572185	13986	4226
GM12878	CEBPB	Jaspar	MA0466.1	wgEncodeEH003212	1342548	5786	493
GM12878	CTCF	Jaspar	MA0139.1	wgEncodeEH001851	565933	55551	46260
GM12878	E2F4	Jaspar	MA0470.1	wgEncodeEH002867	173646	3440	1424
GM12878	EGR1	Jaspar	MA0162.2	wgEncodeEH002328	1060314	16331	17629
GM12878	ELF1	Jaspar	MA0473.1	wgEncodeEH001617	1026618	23008	9029
GM12878	ELK1	Jaspar	MA0028.1	wgEncodeEH002851	100691	5584	2210
GM12878	ETS1	Jaspar	MA0098.2	wgEncodeEH001564	1319961	4120	1416
GM12878	FOS	Jaspar	MA0476.1	wgEncodeEH000622	762222	2239	94
GM12878	JUND	Jaspar	MA0491.1	wgEncodeEH000639	717223	2472	2012
GM12878	MAX	Jaspar	MA0058.2	wgEncodeEH002806	855374	12542	1583
GM12878	MEF2A	Jaspar	MA0052.2	wgEncodeEH001565	3210613	17605	6838
GM12878	MYC	Jaspar	MA0147.2	wgEncodeEH000547	614797	3690	793
GM12878	NFE2	Jaspar	MA0501.1	wgEncodeEH001808	796063	772	33
GM12878	NFYA	Jaspar	MA0060.2	wgEncodeEH002064	428913	1841	1396
GM12878	NFYB	Jaspar	MA0502.1	wgEncodeEH002065	470725	13295	11480
GM12878	NRF1	Jaspar	MA0506.1	wgEncodeEH001846	137117	5683	8533
GM12878	REST	Jaspar	MA0138.2	wgEncodeEH002314	629168	6906	3754
GM12878	PU1	Jaspar	MA0080.3	wgEncodeEH001476	2040890	42938	39126
GM12878	RAD21	Jaspar	MA0139.1	wgEncodeEH000749	565933	33085	29475
GM12878	RFX5	Jaspar	MA0510.1	wgEncodeEH001810	629248	4341	943
GM12878	SIX5	Jaspar	MA0088.1	wgEncodeEH001542	1032447	4839	1872
GM12878	SMC3	Jaspar	MA0139.1	wgEncodeEH001833	565933	30517	27056
GM12878	SP1	Jaspar	MA0079.3	wgEncodeEH001496	1797400	18248	8151
GM12878	SRF	Jaspar	MA0083.2	wgEncodeEH001464	1024023	8544	3651
GM12878	STAT1	Jaspar	MA0137.3	wgEncodeEH001852	1272026	1769	67
GM12878	STAT5A	Jaspar	MA0519.1	wgEncodeEH002321	1292097	7423	335

Cell	Factor	PFM Repository	PFM ID	ChIP-seq ID	# Motifs	# Peaks	# Motifs with Peaks
GM12878	TR4	Jaspar	MA0504.1	wgEncodeEH000697	825980	1263	475
GM12878	USF2	Jaspar	MA0526.1	wgEncodeEH001812	759040	9022	6480
GM12878	YY1	Jaspar	MA0095.2	wgEncodeEH000695	1325447	2077	1333
GM12878	ZBTB33	Jaspar	MA0527.1	wgEncodeEH001488	82928	2144	1919
GM12878	ZNF143	Jaspar	MA0088.1	wgEncodeEH001853	1032447	20024	2866
H1hesc	ATF3	Jaspar	MA0093.2	wgEncodeEH001566	691899	4804	2774
H1hesc	BACH1	Transfac	M00495	wgEncodeEH002842	614421	11457	3442
H1hesc	BRCA1	Jaspar	MA0133.1	wgEncodeEH002801	333055	2025	15
H1hesc	CEBPB	Jaspar	MA0466.1	wgEncodeEH002825	1342548	15557	10788
H1hesc	CTCF	Jaspar	MA0139.1	wgEncodeEH001649	565933	54070	46540
H1hesc	EGR1	Jaspar	MA0162.2	wgEncodeEH001538	1060314	8743	8515
H1hesc	FOSL1	Jaspar	MA0477.1	wgEncodeEH001660	699220	1111	127
H1hesc	GABP	Jaspar	MA0062.2	wgEncodeEH001534	181503	5652	3020
H1hesc	JUN	Jaspar	MA0488.1	wgEncodeEH001854	832374	2148	982
H1hesc	JUND	Jaspar	MA0491.1	wgEncodeEH002023	717223	9550	7368
H1hesc	MAFK	Jaspar	MA0496.1	wgEncodeEH002828	1221488	11425	9083
H1hesc	MAX	Jaspar	MA0058.2	wgEncodeEH001757	855374	11124	3558
H1hesc	MYC	Jaspar	MA0147.2	wgEncodeEH002795	614797	4551	1507
H1hesc	NRF1	Jaspar	MA0506.1	wgEncodeEH001847	137117	4513	7914
H1hesc	P300	Jaspar	MA0243.1	wgEncodeEH001574	600191	8937	381
H1hesc	POU5F1	Jaspar	MA0142.1	wgEncodeEH001636	2201678	3994	3283
H1hesc	RAD21	Jaspar	MA0139.1	wgEncodeEH001836	565933	55674	46626
H1hesc	REST	Jaspar	MA0138.2	wgEncodeEH001498	629168	13269	6981
H1hesc	RFX5	Jaspar	MA0510.1	wgEncodeEH001835	629248	1695	780
H1hesc	RXRA	Jaspar	MA0512.1	wgEncodeEH001560	1110004	1306	337
H1hesc	SIX5	Jaspar	MA0088.1	wgEncodeEH001528	1032447	3422	1929
H1hesc	SP1	Jaspar	MA0079.3	wgEncodeEH001529	1797400	15103	7702
H1hesc	SP2	Jaspar	MA0516.1	wgEncodeEH002302	1587339	2469	1784
H1hesc	SP4	Uniprobe	UP00002	wgEncodeEH002317	503235	5752	2538
H1hesc	SRF	Jaspar	MA0083.2	wgEncodeEH001533	1024023	5102	5466
H1hesc	TBP	Jaspar	MA0108.1	wgEncodeEH001848	834532	17194	564
H1hesc	TCF12	Jaspar	MA0521.1	wgEncodeEH001531	893836	7829	2494
H1hesc	USF1	Jaspar	MA0093.2	wgEncodeEH001532	691899	26028	25899
H1hesc	USF2	Jaspar	MA0526.1	wgEncodeEH001837	759040	6952	6169
H1hesc	YY1	Jaspar	MA0095.2	wgEncodeEH001567	1325447	18310	7308
H1hesc	ZNF143	Jaspar	MA0088.1	wgEncodeEH002802	1032447	30687	4204

References

Buenrostro, J. D., Wu, B., Litzenburger, U. M., Ruff, D., Gonzales, M. L., Snyder, M. P., Chang, H. Y., and Greenleaf, W. J. (2015). Single-cell chromatin accessibility reveals principles of regulatory variation. *Nature*, 523(7561):486–90.

Encode Consortium (2012). An integrated encyclopedia of DNA elements in the human genome. *Nature*, 489(7414):57–74.

Lazarovici, A., Zhou, T., Shafer, A., Dantas Machado, A. C., Riley, T. R., Sandstrom, R., Sabo, P. J., Lu, Y., Rohs, R., Stamatoyannopoulos, J. A., and Bussemaker, H. J. (2013). Probing DNA shape and methylation state on a genomic scale with DNase I. *Proceedings of the National Academy of Sciences of the United States of America*, 110(16):6376–81.

Mathelier, A., Zhao, X., Zhang, A. W., Parcy, F., Worsley-Hunt, R., Arenillas, D. J., Buchman, S., Chen, C. Y., Chou, A., Ienasescu, H., Lim, J., Shyr, C., Tan, G., Zhou, M., Lenhard, B., Sandelin, A., and Wasserman, W. W. (2014). JASPAR 2014: An extensively expanded and updated open-access database of transcription factor binding profiles. *Nucleic Acids Research*, 42(D1):D142—-D147.

Matys, V., Kel-Margoulis, O. V., Fricke, E., Liebich, I., Land, S., Barre-Dirrie, A., Reuter, I., Chekmenev, D., Krull, M., Hornischer, K., Voss, N., Stegmaier, P., Lewicki-Potapov, B., Saxel, H., Kel, A. E., and Wingender, E. (2006). TRANSFAC(R) and its module TRANSCompel(R): transcriptional gene regulation in eukaryotes. *Nucl. Acids Res.*, 34(suppl_1):D108–110.

Piper, J., Elze, M. C., Cauchy, P., Cockerill, P. N., Bonifer, C., and Ott, S. (2013). Wellington: A novel method for the accurate identification of digital genomic footprints from DNase-seq data. *Nucleic Acids Research*, 41(21):e201.

Quach, B. and Furey, T. S. (2016). Defcom: analysis and modeling of transcription factor binding sites using a motif-centric genomic footprinter. *Bioinformatics*, 33(7):956–963.

Robasky, K. and Bulyk, M. L. (2011). UniPROBE, update 2011: Expanded content and search tools in the online database of protein-binding microarray data on protein-DNA interactions. *Nucleic Acids Research*, 39(SUPPL. 1).

Schwessinger, R., Suciu, M. C., McGowan, S. J., Telenius, J., Taylor, S., Higgs, D. R., and Hughes, J. R. (2017). Sasquatch: predicting the impact of regulatory snps on transcription factor binding from cell-and tissue-specific dnase footprints. *Genome research*.

Sherwood, R. I., Hashimoto, T., O'Donnell, C. W., Lewis, S., Barkal, A. A., van Hoff, J. P., Karun, V., Jaakkola, T., and Gifford, D. K. (2014). Discovery of directional and nondirectional pioneer transcription factors by modeling DNase profile magnitude and shape. *Nature biotechnology*, 32(2):171–8.

Sung, M. H., Guertin, M. J., Baek, S., and Hager, G. L. (2014). DNase footprint signatures are dictated by factor dynamics and DNA sequence. *Molecular Cell*, 56(2):275–285.

Zhong, S. (2005). Semi-supervised sequence classification with hmms. *International Journal of Pattern Recognition and Artificial Intelligence*, 19(02):165–182.
